# Asymptomatic patients and asymptomatic phases of Coronavirus Disease 2019 (COVID-19): a population-based surveillance study

**DOI:** 10.1093/nsr/nwaa141

**Published:** 2020-06-23

**Authors:** Xueying Zheng, Sihui Luo, Yong Sun, Mingfeng Han, Jian Liu, Liangye Sun, Liangming Zhang, Ping Ling, Yu Ding, Tengchuan Jin, Zhirong Liu, Jianping Weng

**Affiliations:** The First Affiliated Hospital of USTC, Division of Life Science and Medicine, University of Science and Technology of China, Hefei 230001, China; Clinical Research Hospital (Hefei) of Chinese Academy of Science, Hefei 230001, China; The First Affiliated Hospital of USTC, Division of Life Science and Medicine, University of Science and Technology of China, Hefei 230001, China; Clinical Research Hospital (Hefei) of Chinese Academy of Science, Hefei 230001, China; Key Laboratory for Medical and Health of the 13th Five-Year Plan, Anhui Provincial Center for Disease Control and Prevention, Hefei 230601, China; Fuyang No.2 People's Hospital, Fuyang 236015, China; Anqing Hospital Affiliated to Anhui Medical University (Anqing Municipal Hospital), Anqing, 246003, China; Lu’an People's Hospital, Lu’an 237005, China; Department of Infectious Disease, the First Affiliated Hospital of USTC, Division of Life Science and Medicine, University of Science and Technology of China, Hefei 230001, China; The First Affiliated Hospital of USTC, Division of Life Science and Medicine, University of Science and Technology of China, Hefei 230001, China; Clinical Research Hospital (Hefei) of Chinese Academy of Science, Hefei 230001, China; The First Affiliated Hospital of USTC, Division of Life Science and Medicine, University of Science and Technology of China, Hefei 230001, China; Clinical Research Hospital (Hefei) of Chinese Academy of Science, Hefei 230001, China; The First Affiliated Hospital of USTC, Division of Life Science and Medicine, University of Science and Technology of China, Hefei 230001, China; Division of Life Science and Medicine, University of Science and Technology of China, Hefei 230001, China; Key Laboratory for Medical and Health of the 13th Five-Year Plan, Anhui Provincial Center for Disease Control and Prevention, Hefei 230601, China; The First Affiliated Hospital of USTC, Division of Life Science and Medicine, University of Science and Technology of China, Hefei 230001, China; Clinical Research Hospital (Hefei) of Chinese Academy of Science, Hefei 230001, China

**Keywords:** epidemiology, public health, surveillance, asymptomatic, COVID-19, SARS-CoV-2

## Abstract

In this population-based study, we identified 307 confirmed COVID-19 cases from massive surveillance, including 129,551 individuals screened at fever clinics or returning from Hubei and 3710 close contacts of confirmed COVID-19 patients. Among them, 17 patients were asymptomatic at initial clinical assessment. These asymptomatic patients on admission accounted for a small proportion of all patients (5.54%) with relatively weak transmissibility, and the detection rate was 0.35 per 100 close contacts. Moreover, the dynamics of symptoms of the 307 patients showed that the interval from symptom remission to the final negativity of viral nucleic acid was 5.0 days (IQR 2.0 to 11.0 days), with 14 patients (4.56%) having re-detectable viral RNA after discharge. Together, our findings suggested asymptomatic carriers and presymptomatic patients only accounted for a small proportion of COVID-19. Also, the asymptomatic phase in during recovery of COVID-19 urged that negativity in viral RNA is necessary as de-isolation criteria and follow-up is recommended.

## Introduction

In December 2019, the first case of Coronavirus Disease 2019 (COVID-19) caused by a novel coronavirus of SARS-CoV-2 was reported in Wuhan, China. In merely five months, It has now become a global pandemic and caused more than 2.80 million infected cases and 193 thousand deaths(1). The high transmissibility, severity and case fatality of COVID-19 has posted an enormous burden to the healthcare system.

Under the circumstances that there is yet no vaccine available against SARS-CoV-2, we are relying on comprehensive non-pharmaceutical strategies to contain the spread of the virus. Among these strategies, early detection and timely isolation of infected individuals play an essential role(2). Current massive surveillance strategies of infected individuals mostly relied on the detection of relevant symptoms like fever and coughing. However, previous reports have confirmed that the virus could be transmittable by asymptomatic or presymptomatic patients(3-5). Actually fever, as the main target of surveillance at public places, only appeared in 43.8% of the patients on admission according to a retrospective analysis of hospitalized patients(6). Such symptom-based surveillance disease control measures would be undermined by asymptomatic and presymptomatic individuals transmitting SARS-CoV-2. Therefore, there is urgent need to provide a more accurate estimate on their incidence and their clinical and epidemiological profile of these asymptomatic carriers and presymptomatic patients. Previously, the proportion of asymptomatic patients at diagnosis

ORIGINAL UNEDITED MANUSCRIPT

was reported to range between 7% among the targeting tested Icelandic population(7) to 56% in a nursing home(3). Other reports regarding asymptomatic cases are only single or multicentre studies of hospitalized COVID-19 patients, or series case reports(4, 5, 8, 9). A thorough investigation on the incidence, the longitudinal clinical features and outcomes of asymptomatic patients of COVID-19 is still lacking.

Another asymptomatic phase which would impact on clinical decision and public health strategy is the duration of virus shedding after symptom relief in patients of COVID-19. There is evidence that after symptoms relieved a patient still shedded infectious virus particles(10). Other studies showed that virus nucleic acid shedding time in COVID-19 patients could be more than a month from disease onset(11, 12). Moreover, virus RNA was re-detected positive from the samples in some discharged patients during follow-up visits. These patients were asymptomatic, showing no signs of relapse, and had two consecutive negative results of the viral nucleic acid before discharge(13). Although virus nucleic acid shedding does not equate to infectivity or relapse of the disease, clinicians still urged that extended isolation or observation is necessary, even if all the clinical symptoms disappeared(14). However, the duration of such prolonged isolation has not yet reached consensus.

In this study, we aimed to analyze the asymptomatic phases in patients with SARS-CoV-2 infection among a population-based cohort in three cities Fuyang, Anqing and Lu'an in Anhui Province China. We aimed to provide profiles of asymptomatic patients on detection, description of their transmission, clinical characteristic and outcomes; and draw the dynamic picture of symptoms of COVID-19 patients based on follow-ups to reveal the time of asymptomatic virus shedding after symptom remission.

**Results**

**Summary of the study population**

Between January 22 and March 8, 2020, from a population of 17.7 million in the three cities Fuyang, Anqing and Lu'an, a total of 129,551 individuals travelled from Hubei Province or presented at symptom-based surveillance "fever clinics" were investigated and observed. (See Methods for the details of the surveillance system) Among them, 132 were confirmed SARS-CoV-2 infection. 3,710 close contacts of these 132 confirmed cases were traced and observed, and 175 out of them were confirmed SARS-CoV-2 infection. Together, these 307 confirmed cases where isolated immediately upon confirmation. (Figure 1).

Since February 22, no new cases of COVID-19 were reported, and on March 8 all the COVID-19 cases were discharged in all three cities. Therefore, these patients were all the cases incident in this epidemic wave. Table 1 and Table S1 in the Supplemental Materials summarized their clinical characteristics. We found that patients older of age, males, and patients who had preexisting conditions tended to be more severe. As for laboratory findings on admission, along with the escalation of the severity of the disease, patients were more prone to lymphopenia, showing signs of viral infection in their chest computed tomography (CT) imaging, and higher levels of inflammatory indices such as serum C-reactive protein and interleukin-6.

The median of the incubation period of all the investigated patients was 6.0 days (interquartile range [IQR] 3.0 to 10.0 days), and secondary cases had a slightly longer incubation of 7.0 days (4.0 to 12.5 days, $P = 0.047$, Table S1). The dynamics of symptoms by the severity of the disease were summarized in Figure 2. The most common symptoms at initial assessment were fever (80.64%, 247/307) and coughing (62.87%, 193/307). As the main target of symptom-based surveillance, fever lasted for a median of 7.0 days (IQR 3.8 to 10.0 days), but coughing lasted significantly longer, for a median of 14.0 days (IQR 7.0 to 21.0 days). (Figure 2A) Figure 2B showed that the earliest onset of relevant symptoms presented a median of 5.0 days (IQR 2.8 to 8.0 days) before the screening test of SARS-CoV-2 RNA on detection, fever 5.0 days ( IQR 2.0 to 8.0 days), and coughing 4.0 days (IQR 0 to 7.0 days). 3.26% (10/307) of the cases developed a fever after admission to the designated hospitals, and 24.10%(74/307) developed coughing. Overall, >90% of all the patients could be detected with at least one relevant symptom before receiving the screening viral RNA test.

All the patients were treated according to the recommendations in the *Diagnosis and Treatment Protocol for Novel Coronavirus Pneumonia* released by the National Health Commission of the People's Republic of China.(15) (the National Protocol, see Methods). Details of treatments were available in the Supplemental materials (Table S2). One patient died from COVID-19 in our study population. All other patients recovered from COVID-19 feeling approximately the same before the disease. They were discharged based on the criteria noted in the National Protocol. At the end of the study period, 20 (6.51%) of them were finally classified as mild type of COVID-19, 249 (81.11%) as moderate type, 38 (12.38%) as severe

ORIGINAL UNEDITED MANUSCRIPT

type (see Methods for criteria of classification).

**Clinical characteristics of asymptomatic patients on detection**

Among all 307 cases, we identified 17 asymptomatic cases on detection, who presented with no relevant symptoms at the time of the first test of SARS-CoV-2 RNA before isolation. For their exposure history, four of them returned from Wuhan during the 14 days before hospitalization, 13 had close contact with confirmed COVID-19 cases. (Figure 1) Based on these data, the proportion of asymptomatic patients on detection was 5.54% (17/307), and the detection rate was 0.35% (13/3710).

As Table S3 shows, the 17 asymptomatic patients on detection aged 41.46±17.12 years, nine (52.94%) were in their middle ages (35 to 60 years). Ten (58.52%) were female. One (patient 6) of them had gout, and one (patient 14) had hypertension, others reported no previous comorbidities. Two of them were finally categorized as mild type, and fifteen as moderate type, but none as severe type. No deaths were observed among these 17 patients.

To analyze the profile of these asymptomatic patients, we grouped the study population into four groups according to the presence of symptoms on detection: asymptomatic, afebrile but symptomatic, mild fever and moderate/high fever (Table S3). Compared with those afebrile but symptomatic, and those with mild or moderate/high fever on detection, asymptomatic patients on detection were more likely secondary cases (asymptomatic vs afebrile vs slight fever vs moderate/high fever 76.47% vs 51.16% vs 26.92% vs 22.38%, P = 0.001), less likely to progress into severe type of COVID-19 (0% vs 13.95% vs 5.77% vs 17.48%, P = 0.013), and apparently less likely to be admitted to the intensive care unit (0% vs 4.65% vs 4.81% vs 13.29%, P = 0.057). A tendency of higher proportion of women (female%, asymptomatic vs afebrile vs slight fever vs moderate/high fever, 58.52% vs 60.47% vs 37.50% vs 36.36%, P = 0.013) and less coexisting disorders (11.76% vs 11.63% vs 20.19% vs 23.08%, P = 0.366) were noted in asymptomatic and afebrile cases on detection.

During hospitalization, all these asymptomatic patients received antiviral treatments, largely similar to the other three groups (Table S2 and Table S4). But they are less likely in need of antibiotics and glucocorticoids. Laboratory test results were largely similar among the four groups (Table S3).

The median from admission to discharge of the asymptomatic patients on detection was 15.0 days (IQR 13.0 to 21.0 days) but can be as long as 23 days, which was similar to the other three groups. None of them had re-detectable viral nucleic acid during follow-up after discharge. Figure 3 demonstrated the dynamic of symptoms of 17 asymptomatic patients on detection. Eight patients were asymptomatic carriers who remained asymptomatic throughout their disease course, but only two of these carriers showed no signs of SARS-CoV-2 infection in their chest CT imaging throughout the disease course. Nine were presymptomatic on detection, who had symptoms developed later on despite receiving antiviral treatments after admission to the hospital, making their median incubation period 17.0 days (interquartile range, IQR 14.0 to 19.0 days) and their median duration of symptoms was 9.0 days (IQR 4.0 to 12.0 days). The median duration of all 17 patients from the potential exposure to the source of transmission to admission was 15.0 days (IQR 14.0 to 18.0 days).

Interestingly, of the 176 patients (11 asymptomatic and 165 symptomatic on detection tested for SARS-CoV-2 specific antibodies around 10 to 14 days of hospitalization, we found that the asymptomatic patients on detection had significantly lower levels of both IgG (cut-off index [COI], 2.60 [IQR 1.79 to 9.11] vs 17.99 [IQR 5.23 to 40.72], P = 0.008) and IgM (COI, 0.78 [IQR 0.37 to 1.32] vs 2.70 [IQR 0.99 to 6.80], P = 0.004) compared with those symptomatic on admission. (COI above 1.2 defined as positive; Table 2)

By analyzing the transmission and clustering data (Supplemental materials, Figure S1), We found that the asymptomatic patients on admission were in 11 cluster events involving 37 symptomatic patients and 17 asymptomatic patients on detection. Based on these cluster events, we estimated that one symptomatic patient would transmit the infection to a median of 1 person (IQR 0 to 2 persons; maximum eight persons), while the 17 asymptomatic patients transmitted the disease to no one, which probably due to the swift and strict quarantine policy in these cities.

**Duration of the virus nucleic acid shedding asymptomatic phase during recovery**

We found that in the convalescent patients of COVID-19, there was a phase that their symptoms had relieved, but the results for viral RNA tests in their sample were still positive. We investigated the duration of the virus RNA shedding asymptomatic phase, i.e., the interval of relief of all symptoms to the final negativity results of viral nucleic acid, in all the 307 patients. The median duration of virus RNA shedding asymptomatic phase was 5.0 days (IQR 2.0 to 11.0 days) in all patients, 7.0 days (IQR 3.0 to 13.0 days) in mild type ones, 6.0 days (IQR 2.0 to 11.0 days) in moderate type ones, and 4.0 days (IQR 1.0 to 9.0 days) in severe type ones. (Table 3) Notably, 14 patients had redetectable viral nucleic acid in the follow-up

ORIGINAL UNEDITED MANUSCRIPT

tests after discharge. For these 14 patients, the duration of virus RNA shedding asymptomatic phase can be up to 35.5 days (IQR 31.3 to 41.5 days).

**Discussion**

Asymptomatic infection of SARS-CoV-2 can be infectious and may be an important underlying cause of a pandemic(16). There are three types of different asymptomatic status of COVID-19 infection: asymptomatic carriers, presymptomatic patients in their incubation period on detection and the asymptomatic phase in the convalescent patients. In this study, based on population-based surveillance, we found that asymptomatic carriers and presymptomatic patients on detection only accounted for a small proportion of the total SARS-CoV-2 infection in cities borders Hubei during the epidemic between January and March. Under the stringent measures of social distancing, the transmission of the virus from the asymptomatic patients were well controlled. Also, we found that for all the COVID -19 patients, after they turned asymptomatic, it took approximately one week for tests of viral nucleic acid to turn negative in samples from respiratory tracts.

**Characteristics and transmissibility of asymptomatic patients on detection**

Overall, the proportion of asymptomatic patients on detection, including those asymptomatic carriers and presymptomatic patients, was 5.53% in our study, while 52.9% (9/17, overall 2.93%, 9/307) asymptomatic cases became symptomatic after their first viral RNA test. The proportion of asymptomatic infection was significantly higher in our study than that reported from China CDC (5.53% vs 1.2%)(17). We also found that the proportion of presymptomatic patients on detection was higher than their counterparts among mild/moderate COVID-19 cases in Wuhan (2.93% vs 1.63%)(18). But a recent single centre study outside Wuhan suggested the proportion of asymptomatic carriers were 4.4%,(19) similar to our study. Of note, the detection rate of such asymptomatic infection among close contacts was similar to that in Iceland (0.35% vs 0.57 to 0.8%), but the proportion of presymptomatic infections in our study was lower than Iceland (43%) (7) and the USA nursing home (56%) (3). This could be attributed to several reasons. The targeting testing strategy of the population with a high risk of SARS-CoV-2 infection in the Icelandic study and the relatively enclosed environment of the nursing home may overestimate the incidence of presymptomatic infections. Firstly, the data in our study were collected from a population-based surveillance system, which was under a more natural circumstance and minimized the number of missing cases. Moreover, in Anhui where our study was conducted, unprecedented strict measures were taken as the public health response required since January 24, including massive surveillance, travel restriction, and strict social distancing, universal facemask-wearing during necessary outings and cession of public events. These measures, which had been proved effective to contain the spread of the virus (20-22), contributed to reduce the transmission of the infection and reduce the number of asymptomatic infection cases.

All these asymptomatic patients received antiviral therapy after admission to the hospital. As shown in Table S3, such treatment did not significantly change their duration of hospitalization compared to those with symptoms on detection. Without a randomized trial, we could not determine whether the treatment had an impact on the development of symptoms.

Regarding transmissibility of the asymptomatic carriers and presymptomatic patients, some case report or case series studies provided evidence that SARS-CoV-2 virus could spread from such patients (4, 23, 24). Survey of 77 infector-infectee transmission pairs showed that 44% of secondary cases were infected during the index infections' presymptomatic stage (25). A study from Ningbo used a prospective design to follow up the viral load and clinical manifestations of 2,147 close contacts of symptomatic and asymptomatic COVID-19 cases. They concluded that the virus infection rates of close contacts were 6.30% with symptomatic patients and 4.11% with asymptomatic patients, respectively(26). A recent report of the analysis 455 contacts who were exposed to an asymptomatic virus carrier also suggested the infectivity of asymptomatic carriers might be low. (27) In fact, one patient among the 17 asymptomatic patients in our study (Patient 11) was breastfeeding her 2-month old baby after she was infected with SARS-CoV-2, and she did not pass the infection to her baby. Due to the implementation of timely and effective public health interventions, we were not able to compare the transmission rates. However, based on the epidemiological data in our study, we can make a rough estimate that every asymptomatic patient on admission spread the disease to no one even under the same household, while a symptomatic onset patient could spread to a median of one secondary case and a maximum of eight. Collectively, this indicates that transmission from asymptomatic patients can be completely cut down with its lower infectivity and proper disease control measures, such like the ones that were implemented in Anhui and all over China.

Besides, we also found a potential tendency of a higher proportion of female among asymptomatic and afebrile cases on detection, which is consistent with the previous findings in Shanghai and Nanjing(4, 24). None of these asymptomatic patients on detection in our study progressed to the severe type or died, suggesting that they were of less risk of progressing into severe status. In contrast, symptomatic patients with higher fever on

ORIGINAL UNEDITED MANUSCRIPT

detection tend to have a shorter incubation period and increased risk of severe illness. One potential explanation would be patients asymptomatic on detection had a weaker immune response to the infection. Studies have shown that over-activated immune response could be the underlying causes of pulmonary inflammation and extensive pulmonary damage in severe cases, as well as progression and prognosis of the disease.(28-30) In our study we found that serum SARS-CoV-2 neutralizing antibodies and serum inflammatory cytokine interleukin-6 (Table 2 and Table S3) levels were significantly lower in the asymptomatic patients than those symptomatic on detection at the middle of their disease course, which indicated a lower level of immune activity and might explain the difference in disease prognosis. However, we did not have data of the dynamic change in these indices, and the results should be interpreted with caution.

**Asymptomatic status during the convalescent stage**

It is noteworthy that the asymptomatic status in the convalescent stage of a COVID-19 patient could last for seven days in mild type, and five days in moderate type, which accounted for over 80% of the total number of infection in our study. The guidelines among different countries and regions varied, but most of them suggested that patients could be discharged from the hospital when their symptoms relieved, and they should continue home isolation for 14 days(31-34). In China, COVID-19 patients were discharged only if their nucleic acid tests negative for respiratory tract pathogen twice consecutively and were also asked to be home isolated for 14 days after discharge (15). But emerging evidence showed that patients with re-detectable viral nucleic acid after discharge is not uncommon(35, 36). Although redetectable virus nucleic acid might be caused by the limitation of the RT-PCR method used for testing the viral RNA, and there is no evidence showing patients with redetectable virus nucleic acid would spread the infection, it is necessary to reconsider the criteria for discharge and the duration of home isolation post-discharge. In our study, 14 of the patients (4.56%, 14/307) had re-detectable virus RNA during their follow-up. Most of such re-detectable positivity was within two weeks after discharge, turned negative within the next two weeks, and caused no new infection during home isolation. This proportion is similar to that reported by the Korean CDC (3.13%) (35). These findings highlighted the importance of subsequent viral RNA tests during follow-up even if the patients are discharged with negative viral RNA results. But upon proper follow-up strategies and strict social distancing, these patients could be identified, and the risk of transmission could be minimized. Considering the time from symptom remission to viral nucleic acid was not short, and the issue of re-detectable positivity in our study, the current two-week and four-week follow-up schedule with virus tests after a viral negative discharge is appropriate, provided testing capacity is sufficient.

**Fever was not enough as a solitary indicator for surveillance**

Currently, temperature surveillance of fever at public places was the sole focus of infection-control strategies in some areas. In this study, we found that fever was neither the most frequent nor the symptom with the longest duration though our investigation of symptom dynamics: 83.71% of the cases had a fever at any time of their whole disease duration, but 86.97% cases had coughing. Fever lasted significantly shorter than coughing (7.0 days [IQR 4.0 to 10.0 days] vs 14.0 days [IQR 7.0 to 19.5 days], Figure 2A and 2C). Also, Figure 2B demonstrated that coughing, and perhaps in a combination of other symptoms, could develop earlier than fever. These data indicated that using fever as the only indicator to identify SARS-CoV-2 potential infection is far from sufficient. But continuous massive surveillance or lock-down would be disastrous to the social-economic status and in turn harm the disease control implementation. Together with the two asymptomatic phases discussed above, close tracing of contacts of confirmed cases, strict social distancing, universal facemask wearing would be a more feasible choice before effective vaccine emerges.

**Strengths and Limitations of this study**

This is the first population-based surveillance study. Not only do we provide estimates for the proportion of COVID-19 patients in different asymptomatic phases, but also the dynamics of symptom in different disease severity category and their clinical outcomes. All these results provided evidence for developing strategies for disease control and treatment.

Our study has some limitations. Due to the limited sample size and the strict measures taken to contain the spread of the disease, we could not quantify the relative contributions of asymptomatic or presymptomatic patients to SARS-CoV-2 transmission. Secondly, the proportion of asymptomatic infection (including latent infection) was determined by population-based monitoring. The population-wide seroepidemiological survey has not yet been conducted. Therefore, further study is warranted to gain a better understanding.

**Conclusion**

Asymptomatic carriers and presymptomatic patients only accounted for a small proportion of COVID-19. Massive surveillance and close contact tracing could help to detect these carriers and presymptomatic patients. Asymptomatic status in the convalescent stage of a COVID-19 could last up to a week, indicating negativity in viral RNA is necessary as de-isolation criteria, and that follow-up is recommended.

ORIGINAL UNEDITED MANUSCRIPT

**Methods**

**Study oversight**

This is an observational cohort study. This study is part of the project of "Construction of a bio-information platform for novel coronavirus pneumonia (COVID-19) patients follow-up in Anhui" (ChiCTR2000030331). This study was approved by the institutional board of the First Affiliated Hospital of University of Science and Technology of China (2020-XG(H)-009).

**Population-based surveillance system**

Since the domestic spread of SARS-CoV-2 in January 2020, strict precautionary measures had been implemented in Anhui province by the joint effort of the local governments, the centres for disease control and prevention (CDC), the health commissions, and the communities. These measures included setting up "fever clinics" dedicated to treating patients who presented with fever or any COVID-19 like symptoms(37). In the fever clinics, all patients received tests for SARS-CoV-2 viral nucleic acid and chest CT scan. Any case with positivity in viral nucleic or chest CT abnormality would be admitted to designated hospitals for COVID-19 treatment and isolated in a single ward. Also, massive surveillance was implemented. The individuals fulfilled one of the following criteria were defined as suspected cases and were traced: (1) travel history to Wuhan or Hubei Province during the past 14 days; (2) wild animal exposure during the past 14 days; (3) presentation of COVID-19 like symptoms such as fever, dry coughing, dyspnea; and diarrhoea; (4) close contact with confirmed or suspected COVID-19 patients within two weeks before their disease onset; (5) other potentially suspected cases. All these individuals were identified by community or CDC staff in person or via telephone, subjected to epidemiological investigation. They were home isolated for medical observation and had SARS-CoV-2 viral nucleic acid tested every three days. Similarly, any case with positivity in viral nucleic acid was admitted to the designated hospitals. Others continued to be observed until the $14^{th}$ day. Such measures ensured that we were able to trace all the potential cases of SARS-CoV-2 infection. The procedures were depicted in the Supplemental materials, Figure S2.

**Study Population**

Based on the population-based surveillance system mentioned above, we acquired the information on the number of all cases who had been screened and confirmed in the cities of Anqing, Lu'an and Fuyang of Anhui Province, China, between January 22 and April 16, 2020. We chose these cities because they were Anhui cities most adjacent to the Hubei Province. In these cities, all the confirmed SARS-CoV-2 infected cases were admitted to the following three designated hospitals: Fuyang No.2 People's Hospital, Anqing Hospital Affiliated to Anhui Medical University (Anqing Municipal Hospital), and Lu'an People's Hospital. We collected data and samples of these confirmed cases of SARS-CoV-2 infection hospitalized in these hospitals during the study period. The investigated individuals all agreed to participate in the study and provided written informed consent.

**Confirmation of SARS-CoV-2 infection**

According to the National Protocol (15), SARS-CoV-2 infection was confirmed by positive results in throat swabs or respiratory specimens of real-time reverse transcription-polymerase chain reaction (RT-PCR) assay repeated twice using SARS-CoV-2 nucleic acid detection kits.

In Anhui province, to improve the quality of the detection, a two-step confirmation strategy was adopted. Samples of an individual were first tested in the laboratory of municipal CDC with two different detection kits. The municipal CDC laboratory crosschecked the results from the two kits to report a positive case. Then these positive samples were sent to the laboratory of Anhui Provincial CDC using the same procedure to test for the viral nucleic acid If the positivity could be repeated in the provincial CDC laboratory, this case was finally confirmed as positive. All suspected and confirmed cases in Anhui Province were required to go through such two-step confirmation.

**Data and sample collection**

We collected epidemiological and clinical data of all confirmed cases in the participating hospitals onto case report forms adapted from International Severe Acute Respiratory and Emerging Infection Consortium (ISARIC) / World Health Organization (WHO) Clinical Characterization Protocol for severe emerging infections10. Briefly, information on symptoms and disease onset, potential exposure to the pathogen, visits to healthcare facilities, hospitalization, treatment, pathogen and laboratory tests, and clinical outcomes, and follow-up visits were collected. Notably, for all the confirmed cases, we comprised a daily log at approximately the same time every morning to document the severity of their COVID-19 related symptoms based on the data extracted from the medical record.

Trained investigators collected information from the medical record system and uploaded to the REDCap electronic data capture tools securely hosted at the Division of Life Science and Medicine, University of Science and Technology of China. A second investigator verified these records.

ORIGINAL UNEDITED MANUSCRIPT

Then a third investigator validated the data by crosschecking with the record in the medical record system and communication with the physicians attending the individuals, or telephone interview of the individuals when necessary. Two independent licensed radiologists reviewed the original images of the chest CT scans. We relied on those reports which had consistent interpretation.

We collected serum samples from confirmed cases in the participating hospitals during their hospitalization.

**Classification and treatment**

All the confirmed COVID-19 patients admitted to the participating hospital were attended according to the National Protocol.(15) They were classified into mild, moderate and severe type of COVID-19 based on severity. Mild cases were defined as "the clinical symptoms were mild, and there was no sign of pneumonia on imaging" in align with the National Protocol. Moderate cases were those "showing fever and respiratory symptoms with radiological findings of pneumonia". Severe cases in our study were a combination of severe cases and critical cases defined by the National Protocol.

**Discharge and Follow-up**

A patient should meet all the following criteria according to the National Protocol to be discharged from the designated hospital. (1) body temperature returned to normal for more than three days; (2) significant improvement of respiratory symptoms; (3) significant improvement in pulmonary imaging; (4) samples from respiratory tract were negative twice for SARS-CoV-2 nucleic acid (sampling interval being at least 24 hours).

After discharge, all patients were subjected to home isolation for another 14 days. We followed up these patients two weeks and four weeks after discharge. Fourteen of them had re-detectable viral nucleic acid in their nasopharyngeal swab samples during home isolation, and all turned negative at the end of the fourth week after discharge.

**Definitions**

We defined incubation period as the period between the earliest exposure to the potential transmission source of SARS-CoV-2 to the onset of illness (the presence of the earliest symptom).

Suspected cases were defined in align with the criteria used in the massive surveillance. Confirmed cases were patients who were confirmed SARS-CoV-2 infection through the two-step protocol as described above. Asymptomatic cases on detection were confirmed cases without presence of any relevant symptoms at the first test (i.e., screening test) of SARS-CoV-2 RNA, including two types of patients: asymptomatic carriers and presymptomatic patients on detection. Asymptomatic carriers, i.e., patients with asymptomatic infection, were confirmed cases without any relevant symptoms, with/without a change in chest CT throughout the their disease course (infection) until their SARS-CoV-2 RNA turning negative. Presymptomatic patients on detection were patients who were asymptomatic at their screening test of SARS-CoV-2 but later developed relevant symptoms during hospitalization. We defined asymptomatic phases as the period of time when a confirmed case who presented with no relevant symptoms, which could refer to the time between exposure to transmission source and the onset of symptom(s), or the time between overall symptom relief to the final conversion to negativity in viral RNA tests during recovery, or the duration of SARS-CoV-2 nucleic acid positivity in an asymptomatic carrier.

Based on the presence of fever on detection, we categorized the patients with symptomatic onset into three groups: afebrile, mild fever and moderate/high fever. Patients were considered afebrile on detection if their body temperature stayed under 37.3℃ before the first test of SARS-CoV-2 test. Mild fever was defined as patients who had any record of body temperature above 37.3℃, but never higher than 38.0℃ before detection. Moderate/high fever was defined as patients who had any body temperature record above 38.0℃ before detection.

**SARS-CoV-2 specific antibody detection**

Serum SARS-CoV-2 specific antibody levels were measured with chemiluminescent kits (Kangrun Biotech) for IgA (Doc no. KR/CE-01-B10, Revision A/0), IgG (Doc no. KR/CE-02-B10, Revision A/0) and IgM (Doc no. KR/CE-03-B10, Revision A/0). Briefly, the N-protein or receptor-binding domain (RBD) viral antigens were coated to magnetic particles to catch SARS-CoV-2 specific IgA, IgM and IgG in patient sera. Then a second antibody that recognizes IgA, IgM or IgG was added for detection of IgA, IgM and IgG, respectively. The detected chemiluminescent signal over the background signal was calculated as relative light units (RLU), COI was the ratio of RLU to statistically determined cut-off (criterion). Then RLU was measured using a fully automatic chemical luminescent immunoanalyzer, Kaeser 1000 (Kangrun Biotech, Guangzhou, China).

**Statistical analysis**

Continuous variables were presented as mean ± standard deviation or median (IQR). Comparisons between groups were performed with the Student's t-test, one-way ANOVA, or Mann-Whitney U test when appropriate. Categorical variables were presented as number (%) and compared

ORIGINAL UNEDITED MANUSCRIPT

using the $\chi^2$ test or Fisher's exact test when appropriate. A two-sided α of less than 0.05 was considered statistically significant. Data were analyzed using the R software, version 3.6.1 (R Foundation for Statistical Computing).

**Acknowledgement**

We thank the patients for donating their data and sample. We thank the physicians and administrative staff for their assistance in data collection.

**Author contribution**

XZ contributed in conceptualization, study design, data collection and interpretation, and wrote the first draft. SL contributed to data and sample collection, management and interpretation, and co-wrote the first draft. XZ and SL contributed equally. YS, MH, CH, LS and LZ contributed in data collection and interpretation, administrative support, and critical discussion of the manuscript. LP, YD, TJ and SW contributed in data collection, analysis and interpretation, and discussion of the manuscript. ZL contributed in data interpretation, administrative support, and discussion of the manuscript. JW contributed in conceptualization, study design, funding acquisition, administrative support, data interpretation and critical discussion of the manuscript.

**Funding**

This study is funded by the Fundamental Research Funds for the Central Universities (Grant number. YD9110004001, YD9110002002, and YD9110002008) and AnHui Province Emergency funding for COVID-19 disease (Grant number: 202004a07020002 and 202004a07020004).

**Conflict of interest**

We declare no conflict of interest.

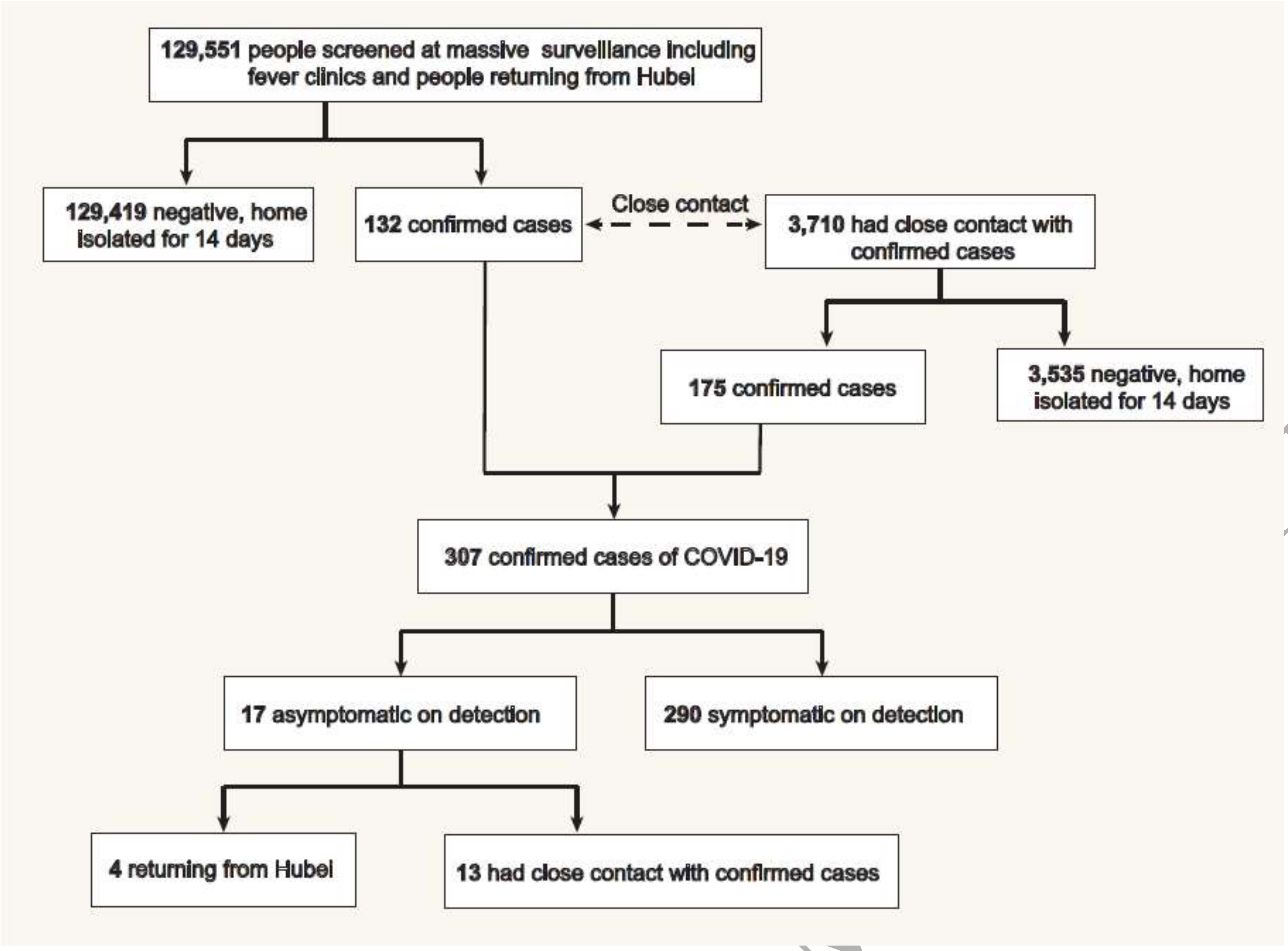

**Figure 1.** The flow of confirmed COVID-19 case ascertainment.

ORIGINAL UNEDITED MANUSCRIPT

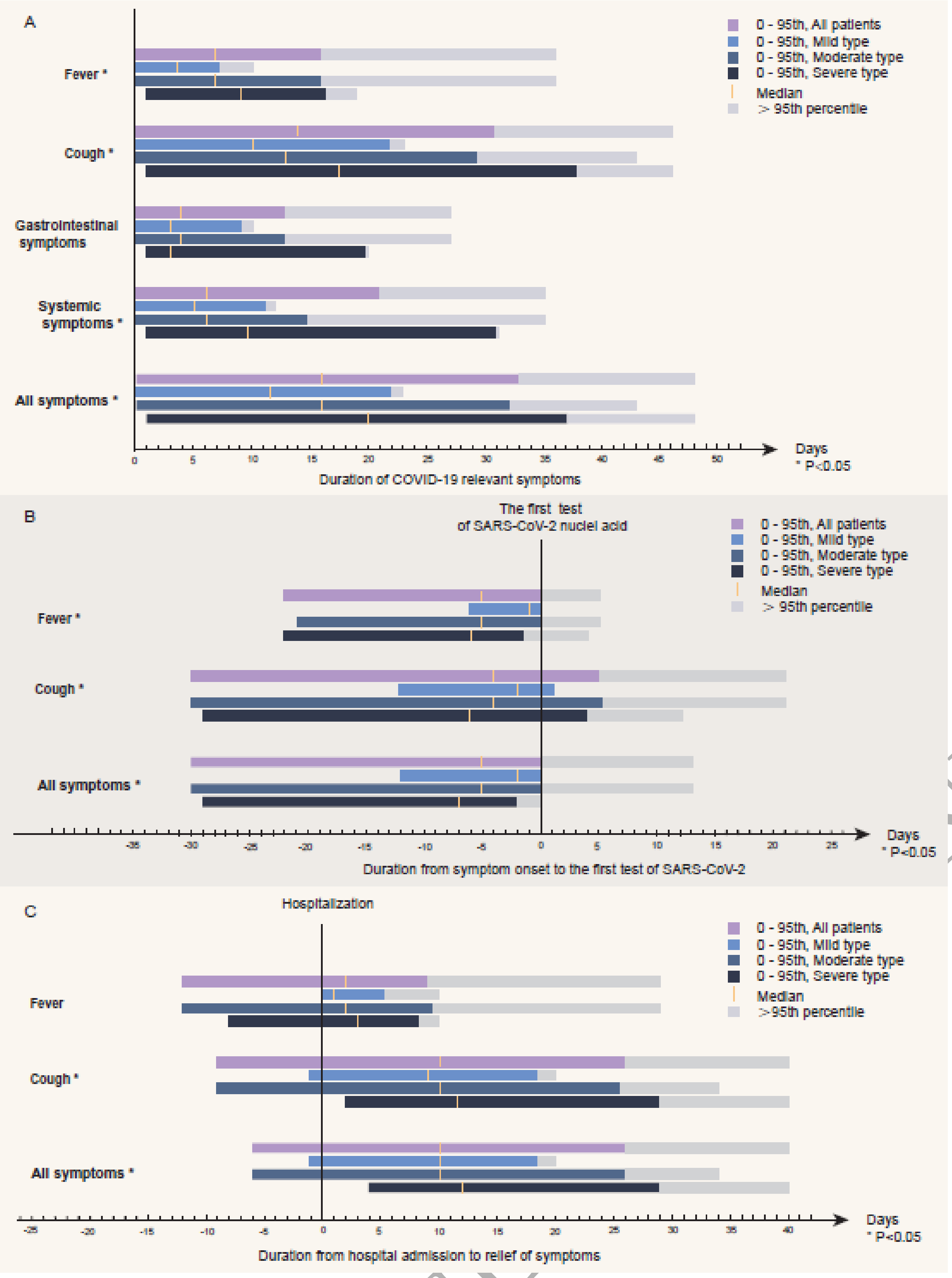

**Figure 2.** Dynamics of COVID-19 relevant symptoms in the study population.

Figure 2 summarizes the dynamics of symptoms by the severity of COVID-19. (A) Overall, patients of more severe illness were prone to have longer duration of fever, coughing and systemic symptoms. For all patients, fever lasted for a median of 7.0 days (IQR 3.8 to 10.0 days), but coughing lasted significantly longer, for a median of 14.0 days (IQR 7.0 to 21.0 days). (B) The earliest onset of relevant symptoms presented a median of 5.0 days (IQR 2.8 to 8.0 days) before the screening test of SARS-CoV-2 RNA on detection, fever 5.0 days (IQR 2.0 to 8.0 days), and coughing 4.0 days (IQR 0 to 7.0 days). 3.26% (10/307) of the cases developed a fever after admission to the hospitals and 24.10%(74/307) developed coughing. (C) All COVID-19 symptoms lasted for a median of 10.0 days (IQR 6.0 to 15.0 days) after admission to the hospitals. But compared with cough (median 10.0 days, IQR 5.0 to 15.0 days) and other symptoms, fever lasted for a significantly shorter duration (median 2.0 days, IQR 0 to 4.0 days).

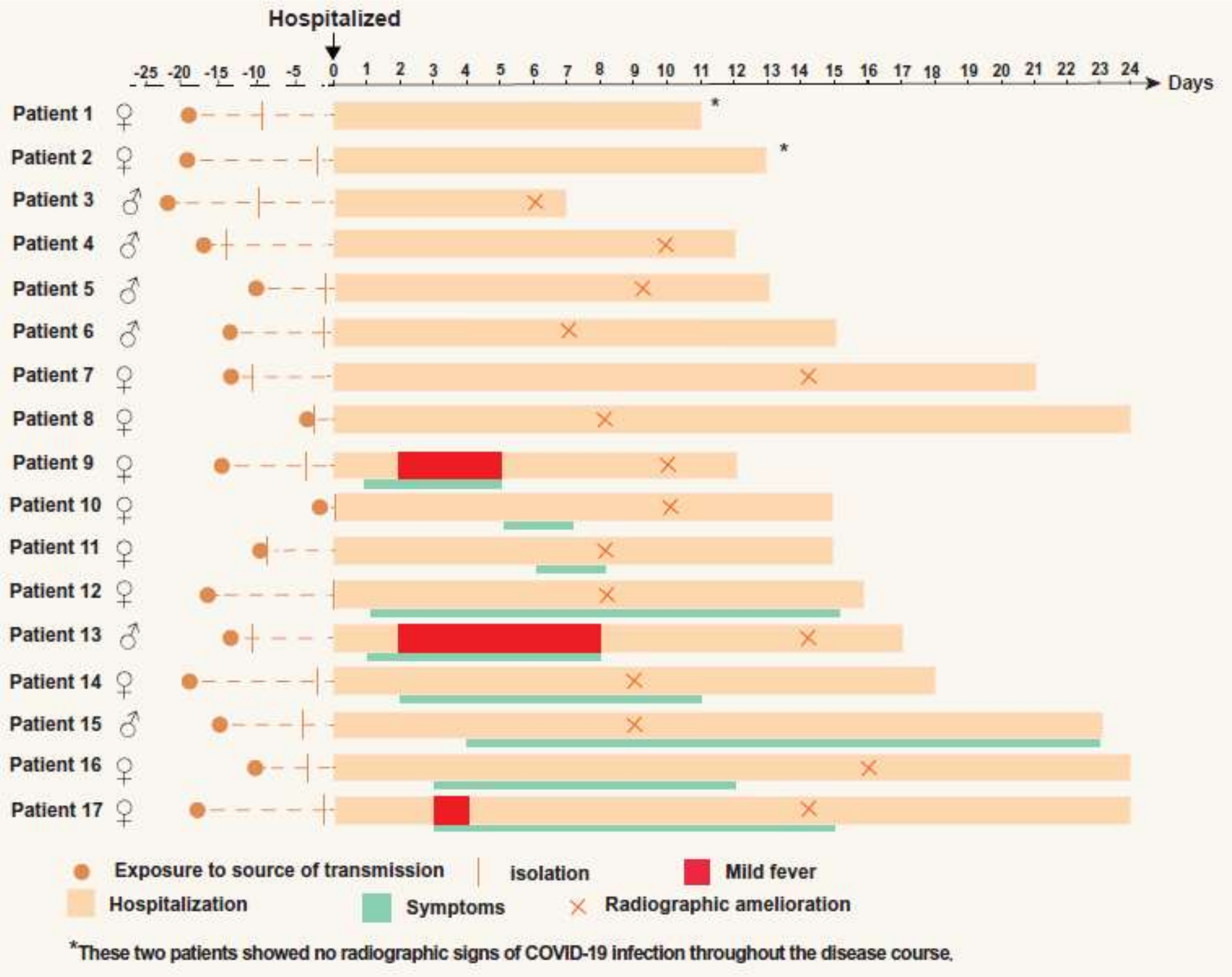

**Figure 3.** Dynamics of symptoms of patients of COVID-19 asymptomatic on detection.

Figure 3 summarizes the dynamics of symptoms related to their epidemiological history, hospitalization and change in their chest computed tomography imaging of the 17 patients asymptomatic when they first received the SARS-CoV-2 nucleic acid test. Eight of them presented with no symptoms throughout their disease course (Patient 1 - 8), and two of them show no radiographic signs of COVID-19 infection at all (Patient 1 and 2). The rest of the nine patients ( Patients 9 - 17) had a median of incubation period of 17.0 days (IQR 14.0 to 19.0 days). All these 17 patients had were hospitalized for a median of 15.0 days (IQR 13.0 to 21.0 days).

ORIGINAL UNEDITED MANUSCRIPT

**Table 1. Clinical and epidemiological characteristics of the confirmed cases of COVID-19 by the severity of the disease**.

| | All patients | Mild | Moderate | Severe | P value |
|---|---|---|---|---|---|
| N | 307 | | | | |
| **Clinical and epidemiological Characteristics** | | | | | |
| Age, years | 42.90±14.57 | 26.67±12.69 | 42.50±13.35 | 54.04±14.46 | 0.001 |
| 0-14, n(%) | 8(2.61) | 4(20.00) | 4(1.61) | 0 | 0.002 |
| 15-49, n(%) | 203(66.12) | 16(80.00) | 170(68.27) | 17(44.74) | |
| 50-64, n(%) | 76(24.76) | 0 | 65(26.10) | 11(28.95) | |
| ≥65, n(%) | 20(6.51) | 0 | 10(4.02) | 10(26.32) | |
| Male sex, n(%) | 180(58.63) | 5(25.00) | 147(59.04) | 28(73.68) | 0.002 |
| Current smoking, n(%) | | | | | 0.855 |
| Current smoker | 27(8.79) | 1(5.00) | 22(8.84) | 4(10.53) | |
| Former smoker or never smoked | 280(91.21) | 19(95.00) | 227(91.16) | 34(89.47) | |
| Exposure to source of transmission within the past 14 days, n(%) | | | | | 0.007 |
| Recently visited Wuhan | 132(43.00) | 9(45.00) | 114(45.78) | 9(23.68) | |
| Had contacted with confirmed patients | 95(30.94) | 8(40.00) | 75(30.12) | 12(31.58) | |
| Not clear | 80(26.06) | 3(15.00) | 60(24.10) | 17(44.74) | |
| Coexisting disorder, n(%) | 61(19.87) | 1(5.00) | 42(16.87) | 18(47.37) | 0.001 |
| Diabetes | 11(3.58) | 0 | 3(1.20) | 8(21.05) | 0.001 |
| Hypertension | 36(11.73) | 1(5.00) | 23(9.24) | 12(31.58) | 0.001 |
| Cardiovascular disease | 6(1.95) | 0 | 4(1.61) | 2(5.26) | 0.210 |
| Chronic pulmonary disease | 6(1.95) | 0 | 3(1.20) | 3(7.89) | 0.051 |
| Chronic liver disease | 13(4.23) | 0 | 11(4.42) | 2(5.26) | 0.358 |
| Chronic renal disease | 2(0.65) | 0 | 2(0.80) | 0 | 1.000 |
| Rheumatic disease | 3(0.98) | 0 | 2(0.80) | 1(2.63) | 0.468 |
| Symptoms, n (%) | | | | | |
| Fever | 247(80.46) | 14(70.00) | 201(80.72) | 32(84.21) | 0.409 |
| Coughing | 193(62.87) | 12(60.00) | 154(61.85) | 27(71.05) | 0.132 |
| Sputum production | 98(31.92) | 5(25.00) | 81(32.53) | 12(31.58) | 0.260 |
| Hemoptysis | 2(0.65) | 0 | 2(0.80) | 0 | 0.173 |
| Sore throat | 15(4.89) | 2(10.00 ) | 11(4.42) | 2(5.26) | 0.194 |
| Snivel | 10(3.26) | 2(10.00 ) | 7(2.81) | 1(2.63) | 0.179 |
| Gasp | 6(1.95) | 0 | 4(1.61) | 2(5.26) | 0.336 |
| Dyspnea | 4(1.30) | 0 | 2(0.80) | 2(5.26) | 0.204 |
| Headache | 15(4.89) | 2(10.00 ) | 12(4.82) | 1(2.63) | 0.559 |
| Myalgia | 20(6.51) | 2(10.00 ) | 12(4.82) | 1(2.63) | 0.498 |
| arthralgia | 1(0.33) | 0 | 1(0.40) | 0 | 0.649 |
| Fatigue | 47(15.31) | 6(30.00) | 38(15.26) | 3(7.89) | 0.131 |
| Gastrointestinal symptoms | 23(7.49) | 0 | 21(8.43) | 2(5.26) | 0.485 |
| Admission to ICU, n (%) | 27(8.79) | 0 | 0 | 27(71.05) | 0.001 |
| Median (IQR) time from onset of symptom to admission, days | 4.5(2.0,7.0) | 3.00(1.00,3.75) | 4.00(2.00,7.00) | 7.00(4.25,10.00) | 0.001 |
| Median (IQR) time from onset of symptom to discharge, days | 22.0(18.0,27.0) | 17.00(16.00,23.75) | 21.00(18.00,26.00) | 26.00(22.00,30.00) | 0.001 |
| Median (IQR) time from admission to discharge, days | 16.0(13.0,20.0) | 14.50(12.00,20.25) | 16.00(13.00,20.00) | 17.50(14.25,20.00) | 0.189 |
| Median (IQR) incubation period, days | 6.0(3.0,10.0) | 4.00(1.75,9.25) | 6.00(3.00,10.50) | 2.00(1.00,4.75) | 0.040 |
| **Laboratory findings on admission** (mean±SD unless otherwise noted) | | | | | |
| $SaO_2$, % | 97.66±1.83 | 98.05±0.69 | 97.94±0.96 | 95.66±4.08 | 0.003 |
| White blood cell count, × $10^9$/L | 5.28±2.17 | 5.64±2.63 | 5.07±1.91 | 6.46±3.2 | 0.022 |
| ＜4 (leucopenia) ), n (%) | 93(30.29) | 4(20.00) | 79(31.73) | 10(26.32) | 0.077 |
| Neutrophil percentage,% | 65.24±13.34 | 53.77±16.69 | 64.66±11.78 | 74.99±15.01 | 0.001 |
| Lymphocyte percentage, % | 24.43±10.65 | 30.15±12.45 | 25.30±10.20 | 15.83±7.86 | 0.001 |
| ＜20 (lymphopenia), n (%) | 120(39.09) | 4(20.00) | 87(34.94) | 29(76.32) | 0.001 |

ORIGINAL UNEDITED MANUSCRIPT

| | | | | | |
|---|---|---|---|---|---|
| Hemoglobin, g/L | 135.84±15.88 | 128.20±12.38 | 136.99±15.48 | 132.41±18.65 | 0.853 |
| Platelet count, × $10^9$/L | 184.82±76.11 | 203.40±65.53 | 183.12±75.49 | 186.05±85.42 | 0.286 |
| PT, s | 12.19±2.44 | 12.04±1.24 | 12.14±2.60 | 12.64±1.47 | 0.214 |
| APTT, s | 33.95±8.66 | 38.23±7.97 | 34.36±8.76 | 29.66±6.72 | 0.004 |
| ALT, U/L, median (IQR) | 24.00(15.00,37.00) | 13.50(8.75,18.00) | 25.00(15.00,38.00) | 27.50(18.50,40.75) | 0.001 |
| AST, U/L, median (IQR) | 25.00(20.00,32.00) | 20.00(17.00,25.25) | 25.00(20.00,33.00) | 29.00(23.00,38.50) | 0.003 |
| Total bilirubin, mmol/L | 13.29±7.74 | 12.81±8.11 | 13.05±7.30 | 15.10±9.96 | 0.580 |
| Creatinine, umol/L | 64.42±16.52 | 52.43±14.51 | 65.15±16.71 | 66.23±13.83 | 0.002 |
| BUN, mmol/L | 4.23±1.82 | 3.82±0.88 | 4.15±1.80 | 5.00±2.14 | 0.022 |
| Blood glucose, mmol/L | 6.42±2.11 | 5.69±1.49 | 6.15±1.50 | 8.61±3.91 | 0.001 |
| Procalcitonin, ng/mL, median (IQR) | 0.04(0.02,0.07) | 0.04(0.03,0.05) | 0.04(0.02,0.06) | 0.02(0.01,0.11) | 0.899 |
| C reactive protein, mg/L | 11.50(2.60,33.18) | 1.40(0.70,2.60) | 10.70(2.70,28.20) | 39.75(24.18,78.53) | 0.001 |
| CK, U/L, median (IQR) | 60.00(42.00,86.00) | 59.00(34.00,67.00) | 59.00(42.50,87.50) | 73.00(44.00,115.00) | 0.004 |
| CK-MB, U/L, median (IQR) | 7.00(3.00,11.00) | 4.00(3.00,7.50) | 6.50(3.00,11.00) | 9.00(5.00,13.00) | 0.022 |
| Interleukin 6, pg/ml, median (IQR) | 15.50(5.10,31.50) | 4.50(3.10,9.00) | 12.50(4.80,24.00) | 46.20(27.00,71.50) | 0.001 |
| Urinary protein (+ or ++), n(%) | 37(12.05) | 2(10.00) | 31(12.45) | 4(10.53) | 0.124 |
| Abnormalities in chest CT on admission, n (%) | 287(93.49) | 0 | 249(100.00) | 38(100.00) | 0.001 |

**Abbreviations:** IQR interquartile range, $SaO_2$ Saturation of oxygen, PT prothrombin time, APTT activated partial thromboplastin time, ALT alanine aminotransferase, AST aspartate aminotransferase, BUN blood urea nitrogen, CK creatinine kinase, CK-MB creatinine kinase-MB.

ORIGINAL UNEDITED MANUSCRIPT

**Table 2. Levels of serum SARS-CoV-2 neutralizing antibodies in confirmed cases of COVID-19.**

| | All patients | Symptomatic patients on admission | Asymptomatic patients on admission | P value |
|---|---|---|---|---|
| N | 176 | 165 | 11 | – |
| IgG, COI (IQR) | 16.38(4.83,38.83) | 17.99(5.23,40.72) | 2.60(1.79,9.11) | 0.008 |
| IgG (positivity), n (%) | 155(50.49) | 145(50.00) | 10(90.91) | 1.000 |
| IgM, COI (IQR) | 2.43(0.97,6.51) | 2.70(0.99,6.80) | 0.78(0.37,1.32) | 0.004 |
| IgM (positivity), n (%) | 114(37.13) | 110(37.93) | 4(36.36) | 0.053 |
| IgA COI (IQR) | 4.11(1.41,10.07) | 4.18(1.48,10.41) | 2.93(0.98,5.46) | 0.203 |
| IgA (positivity), n (%) | 138(44.95) | 130(44.83) | 8(72.72) | 0.705 |

ORIGINAL UNEDITED MANUSCRIPT

**Table 3. The duration of symptoms of the confirmed cases of COVID-19.**

| Symptoms | All patients | Mild type | Moderate type | Severe type | P values |
|---|---|---|---|---|---|
| Fever, days (IQR) | 7.0(3.8,10.0 ) | 3.5(2.0,5.0) | 7.0(3.0,10.0) | 9.0(7.3,11.8) | 0.001 |
| Cough, days (IQR) | 14.0(7.0,21.0) | 10.0(4.0,15.0) | 13.0(6.0,20.0) | 17.5(14.0,26.3) | 0.001 |
| Gastrointestinal symptoms, days (IQR) | 4.0(2.0,7.0) | 3.0(1.0,6.0) | 4.0(2.0,6.5) | 3.0(1.5,7.0) | 0.782 |
| Systemic symptoms, days (IQR) | 6.0(2.0,10.0) | 5.0(2.8,9.3) | 6.0(2.0,10.0) | 10.0(2.0,21.5) | 0.021 |
| All symptoms, days (IQR) | 16.0(11.0,22.0) | 11.5(7.0,14.0) | 16.0(10.0,21.0) | 20.0(16.0,29.0) | 0.001 |

ORIGINAL UNEDITED MANUSCRIPT
