# Asymptomatic patients and asymptomatic phases of Coronavirus Disease 2019 (COVID-19): a population-based surveillance study

**DOI:** 10.1093/nsr/nwaa141

**Published:** 2020-06-23

**Authors:** Xueying Zheng, Sihui Luo, Yong Sun, Mingfeng Han, Jian Liu, Liangye Sun, Liangming Zhang, Ping Ling, Yu Ding, Tengchuan Jin, Zhirong Liu, Jianping Weng

**Affiliations:** The First Affiliated Hospital of USTC, Division of Life Science and Medicine, University of Science and Technology of China, Hefei 230001, China; Clinical Research Hospital (Hefei) of Chinese Academy of Sciences, Hefei 230001, China; The First Affiliated Hospital of USTC, Division of Life Science and Medicine, University of Science and Technology of China, Hefei 230001, China; Clinical Research Hospital (Hefei) of Chinese Academy of Sciences, Hefei 230001, China; Key Laboratory for Medical and Health of the 13th Five-Year Plan, Anhui Provincial Center for Disease Control and Prevention, Hefei 230601, China; Fuyang No.2 People's Hospital, Fuyang 236015, China; Anqing Hospital Affiliated to Anhui Medical University (Anqing Municipal Hospital), Anqing 246003, China; Lu’an People's Hospital, Lu’an 237005, China; Department of Infectious Disease, the First Affiliated Hospital of USTC, Division of Life Science and Medicine, University of Science and Technology of China, Hefei 230001, China; The First Affiliated Hospital of USTC, Division of Life Science and Medicine, University of Science and Technology of China, Hefei 230001, China; Clinical Research Hospital (Hefei) of Chinese Academy of Sciences, Hefei 230001, China; The First Affiliated Hospital of USTC, Division of Life Science and Medicine, University of Science and Technology of China, Hefei 230001, China; Clinical Research Hospital (Hefei) of Chinese Academy of Sciences, Hefei 230001, China; The First Affiliated Hospital of USTC, Division of Life Science and Medicine, University of Science and Technology of China, Hefei 230001, China; Division of Life Science and Medicine, University of Science and Technology of China, Hefei 230001, China; Key Laboratory for Medical and Health of the 13th Five-Year Plan, Anhui Provincial Center for Disease Control and Prevention, Hefei 230601, China; The First Affiliated Hospital of USTC, Division of Life Science and Medicine, University of Science and Technology of China, Hefei 230001, China; Clinical Research Hospital (Hefei) of Chinese Academy of Sciences, Hefei 230001, China

**Keywords:** epidemiology, public health, surveillance, asymptomatic, COVID-19, SARS-CoV-2

## Abstract

In this population-based study, we identified 307 confirmed COVID-19 cases from massive surveillance, including 129 551 individuals screened at fever clinics or returning from Hubei and 3710 close contacts of confirmed COVID-19 patients. Among them, 17 patients were asymptomatic at initial clinical assessment. These asymptomatic patients on admission accounted for a small proportion of all patients (5.54%) with relatively weak transmissibility, and the detection rate was 0.35 per 100 close contacts. Moreover, the dynamics of symptoms of the 307 patients showed that the interval from symptom remission to the final negativity of viral nucleic acid was 5.0 days (interquartile range 2.0 to 11.0 days), with 14 patients (4.56%) having re-detectable viral RNA after discharge. Overall, our findings suggested asymptomatic carriers and presymptomatic patients only accounted for a small proportion of COVID-19 patients. Also, the asymptomatic phase during recovery from COVID-19 implied that negativity in viral RNA is necessary as a de-isolation criterion and follow-up is recommended.

## INTRODUCTION

In December 2019, the first case of Coronavirus Disease 2019 (COVID-19) caused by a novel coronavirus of SARS-CoV-2 was reported in Wuhan, China. In merely five months, it had become a global pandemic and caused more than 2.8 million infected cases and more than 193 000 deaths [1]. The high transmissibility, severity and case fatality of COVID-19 has put an enormous burden on the healthcare system.

As there is yet no vaccine available against SARS-CoV-2, we are relying on comprehensive non-pharmaceutical strategies to contain the spread of the virus. Among these strategies, early detection and timely isolation of infected individuals play an essential role [2]. Current massive surveillance strategies of infected individuals mostly rely on the detection of relevant symptoms like fever and coughing. However, previous reports have confirmed that the virus could be transmittable by asymptomatic or presymptomatic patients [3–5]. Fever, as the main target of surveillance in public places, actually only appeared in 43.8% of patients on admission, according to a retrospective analysis of hospitalized patients [6]. Such symptom-based surveillance disease control measures would be undermined by asymptomatic and presymptomatic individuals transmitting SARS-CoV-2. Therefore, there is urgent need to provide a more accurate estimate on the incidence, as well as the clinical and epidemiological profile of, these asymptomatic carriers and presymptomatic patients. Previously, the proportion of asymptomatic patients at diagnosis was reported to range between 7% among the targeted and tested Icelandic population [7] to 56% in a nursing home [3]. Other reports regarding asymptomatic cases are only single or multicenter studies of hospitalized COVID-19 patients, or series case reports [4,5,8,9]. A thorough investigation into the incidence, longitudinal clinical features and outcomes of asymptomatic patients of COVID-19 is still lacking.

Another asymptomatic phase which would impact on clinical decision and public health strategy is the duration of virus shedding after symptom relief in patients of COVID-19. There is evidence that after symptom relief a patient still sheds infectious virus particles [10]. Other studies showed that virus nucleic acid shedding time in COVID-19 patients could be more than a month from disease onset [11,12]. Moreover, virus RNA was re-detected as positive from the samples in some discharged patients during follow-up visits. These patients were asymptomatic, showing no signs of relapse, and had two consecutive negative results of the viral nucleic acid before discharge [13]. Although virus nucleic acid shedding does not equate to infectivity or relapse of the disease, clinicians still urged that extended isolation or observation is necessary, even if all the clinical symptoms disappeared [14]. However, the duration of such prolonged isolation has not yet reached consensus.

In this study, we aimed to analyze the asymptomatic phases in patients with SARS-CoV-2 infection among a population-based cohort in three cities: Fuyang, Anqing and Lu’an in Anhui Province, China. We aimed to provide profiles of asymptomatic patients with regard to detection, description of their transmission, clinical characteristics and outcomes, and draw the dynamic picture of symptoms of COVID-19 patients based on follow-ups to reveal the time of asymptomatic virus shedding after symptom remission.

## RESULTS

### Summary of the study population

**Figure 1.**
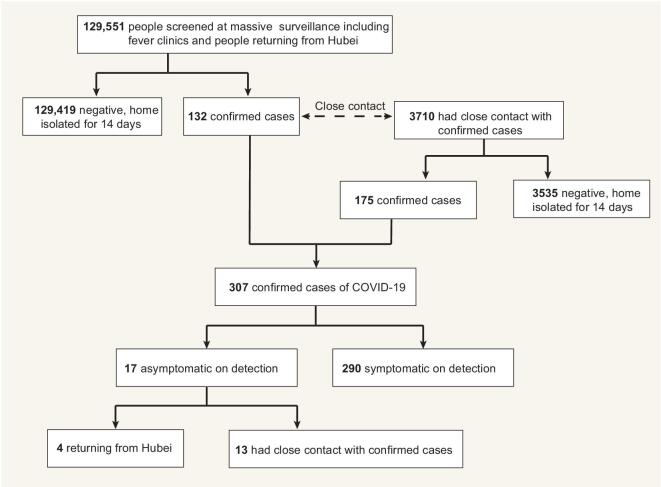
The flow of confirmed COVID-19 case ascertainment. Between 22 January and 8 March 2020, from a population of 17.7 million in the study cities, a total of 129 551 were investigated by the massive surveillance system. Among them, 132 were confirmed SARS-CoV-2 infection. 3710 close contacts of these 132 confirmed cases were traced and observed, and 175 out of those were confirmed as having SARS-CoV-2 infection. Together, these 307 confirmed cases were isolated immediately upon confirmation. Among 307 confirmed cases, 17 presented with no COVID-19 relevant symptoms on detection.

Between 22 January and 8 March 2020, from a population of 17.7 million in the three cities of Fuyang, Anqing and Lu’an, a total of 129 551 individuals who had traveled from Hubei Province or presented at symptom-based surveillance ‘fever clinics’ were investigated and observed (see Methods for details of the surveillance system). Among them, 132 were confirmed as having SARS-CoV-2 infection. Of these 132 confirmed cases, 3710 were traced and observed, and 175 of those were confirmed as having SARS-CoV-2 infection. Together, these 307 confirmed cases were isolated immediately upon confirmation (Fig. 1).

Since 22 February, no new cases of COVID-19 were reported, and on 8 March all COVID-19 cases were discharged in all three cities. Therefore, these patients constituted all the incidents in this epidemic wave. Table 1 and Table S1 in the Supplementary Materials summarize their clinical characteristics. We found that patients older of age, male, and patients who had pre-existing conditions tended to be more severe. As for laboratory findings on admission, along with the escalation of the severity of the disease, patients were more prone to lymphopenia, showing signs of viral infection in their chest computed tomography (CT) imaging, and higher levels of inflammatory indices such as serum C-reactive protein and interleukin-6.

**Table 1.** Clinical and epidemiological characteristics of the confirmed cases of COVID-19 by the severity of the disease.

	All patients	Mild	Moderate	Severe	*P* value
N	307				
**Clinical and epidemiological characteristics**					
Age, years	42.90±14.57	26.67±12.69	42.50±13.35	54.04±14.46	0.001
0–14, n(%)	8(2.61)	4(20.00)	4(1.61)	0	0.002
15–49, n(%)	203(66.12)	16(80.00)	170(68.27)	17(44.74)	
50–64, n(%)	76(24.76)	0	65(26.10)	11(28.95)	
≥65, n(%)	20(6.51)	0	10(4.02)	10(26.32)	
Male sex, n(%)	180(58.63)	5(25.00)	147(59.04)	28(73.68)	0.002
Current smoking, n(%)					0.855
Current smoker	27(8.79)	1(5.00)	22(8.84)	4(10.53)	
Former smoker or never smoked	280(91.21)	19(95.00)	227(91.16)	34(89.47)	
Exposure to source of transmission within the past 14 days, n(%)		0.007			
Recently visited Wuhan	132(43.00)	9(45.00)	114(45.78)	9(23.68)	
Had contacted with confirmed patients	95(30.94)	8(40.00)	75(30.12)	12(31.58)	
Not clear	80(26.06)	3(15.00)	60(24.10)	17(44.74)	
Coexisting disorder, n(%)	61(19.87)	1(5.00)	42(16.87)	18(47.37)	0.001
Diabetes	11(3.58)	0	3(1.20)	8(21.05)	0.001
Hypertension	36(11.73)	1(5.00)	23(9.24)	12(31.58)	0.001
Cardiovascular disease	6(1.95)	0	4(1.61)	2(5.26)	0.210
Chronic pulmonary disease	6(1.95)	0	3(1.20)	3(7.89)	0.051
Chronic liver disease	13(4.23)	0	11(4.42)	2(5.26)	0.358
Chronic renal disease	2(0.65)	0	2(0.80)	0	1.000
Rheumatic disease	3(0.98)	0	2(0.80)	1(2.63)	0.468
Symptoms, n(%)					
Fever	247(80.46)	14(70.00)	201(80.72)	32(84.21)	0.409
Coughing	193(62.87)	12(60.00)	154(61.85)	27(71.05)	0.132
Sputum production	98(31.92)	5(25.00)	81(32.53)	12(31.58)	0.260
Hemoptysis	2(0.65)	0	2(0.80)	0	0.173
Sore throat	15(4.89)	2(10.00)	11(4.42)	2(5.26)	0.194
Snivel	10(3.26)	2(10.00)	7(2.81)	1(2.63)	0.179
Gasp	6(1.95)	0	4(1.61)	2(5.26)	0.336
Dyspnea	4(1.30)	0	2(0.80)	2(5.26)	0.204
Headache	15(4.89)	2(10.00)	12(4.82)	1(2.63)	0.559
Myalgia	20(6.51)	2(10.00)	12(4.82)	1(2.63)	0.498
Arthralgia	1(0.33)	0	1(0.40)	0	0.649
Fatigue	47(15.31)	6(30.00)	38(15.26)	3(7.89)	0.131
Gastrointestinal symptoms	23(7.49)	0	21(8.43)	2(5.26)	0.485
Admission to ICU, n(%)	27(8.79)	0	0	27(71.05)	0.001
Median (IQR) time from onset of symptom to admission, days	4.5(2.0,7.0)	3.00(1.00,3.75)	4.00(2.00,7.00)	7.00(4.25,10.00)	0.001
Median (IQR) time from onset of symptom to discharge, days	22.0(18.0,27.0)	17.00(16.00,23.75)	21.00(18.00,26.00)	26.00(22.00,30.00)	0.001
Median (IQR) time from admission to discharge, days	16.0(13.0,20.0)	14.50(12.00,20.25)	16.00(13.00,20.00)	17.50(14.25,20.00)	0.189
Median (IQR) incubation period, days	6.0(3.0,10.0)	4.00(1.75,9.25)	6.00(3.00,10.50)	2.00(1.00,4.75)	0.040
**Laboratory findings on admission** (mean±SD unless otherwise noted)					
SaO_2_, %	97.66±1.83	98.05±0.69	97.94±0.96	95.66±4.08	0.003
White blood cell count, × 10^9^/L	5.28±2.17	5.64±2.63	5.07±1.91	6.46±3.2	0.022
<4 (leucopenia) ), n(%)	93(30.29)	4(20.00)	79(31.73)	10(26.32)	0.077
Neutrophil percentage,%	65.24±13.34	53.77±16.69	64.66±11.78	74.99±15.01	0.001
Lymphocyte percentage, %	24.43±10.65	30.15±12.45	25.30±10.20	15.83±7.86	0.001
<20 (lymphopenia), n(%)	120(39.09)	4(20.00)	87(34.94)	29(76.32)	0.001
Hemoglobin, g/L	135.84±15.88	128.20±12.38	136.99±15.48	132.41±18.65	0.853
Platelet count, × 10^9^/L	184.82±76.11	203.40±65.53	183.12±75.49	186.05±85.42	0.286
PT, s	12.19±2.44	12.04±1.24	12.14±2.60	12.64±1.47	0.214
APTT, s	33.95±8.66	38.23±7.97	34.36±8.76	29.66±6.72	0.004
ALT, U/L, median (IQR)	24.00(15.00,37.00)	13.50(8.75,18.00)	25.00(15.00,38.00)	27.50(18.50,40.75)	0.001
AST, U/L, median (IQR)	25.00(20.00,32.00)	20.00(17.00,25.25)	25.00(20.00,33.00)	29.00(23.00,38.50)	0.003
Total bilirubin, mmol/L	13.29±7.74	12.81±8.11	13.05±7.30	15.10±9.96	0.580
Creatinine, umol/L	64.42±16.52	52.43±14.51	65.15±16.71	66.23±13.83	0.002
BUN, mmol/L	4.23±1.82	3.82±0.88	4.15±1.80	5.00±2.14	0.022
Blood glucose, mmol/L	6.42±2.11	5.69±1.49	6.15±1.50	8.61±3.91	0.001
Procalcitonin, ng/mL, median (IQR)	0.04(0.02,0.07)	0.04(0.03,0.05)	0.04(0.02,0.06)	0.02(0.01,0.11)	0.899
C reactive protein, mg/L	11.50(2.60,33.18)	1.40(0.70,2.60)	10.70(2.70,28.20)	39.75(24.18,78.53)	0.001
CK, U/L, median (IQR)	60.00(42.00,86.00)	59.00(34.00,67.00)	59.00(42.50,87.50)	73.00(44.00,115.00)	0.004
CK-MB, U/L, median (IQR)	7.00(3.00,11.00)	4.00(3.00,7.50)	6.50(3.00,11.00)	9.00(5.00,13.00)	0.022
Interleukin 6, pg/ml, median (IQR)	15.50(5.10,31.50)	4.50(3.10,9.00)	12.50(4.80,24.00)	46.20(27.00,71.50)	0.001
Urinary protein (+ or ++), n(%)	37(12.05)	2(10.00)	31(12.45)	4(10.53)	0.124
Abnormalities in chest CT on admission, n(%)	287(93.49)	0	249(100.00)	38(100.00)	0.001

**Abbreviations:** IQR, interquartile range; SaO_2_, saturation of oxygen; PT, prothrombin time; APTT, activated partial thromboplastin time; ALT, alanine aminotransferase; AST, aspartate aminotransferase; BUN, blood urea nitrogen; CK, creatinine kinase; CK-MB, creatinine kinase-MB.

**Figure 2.**
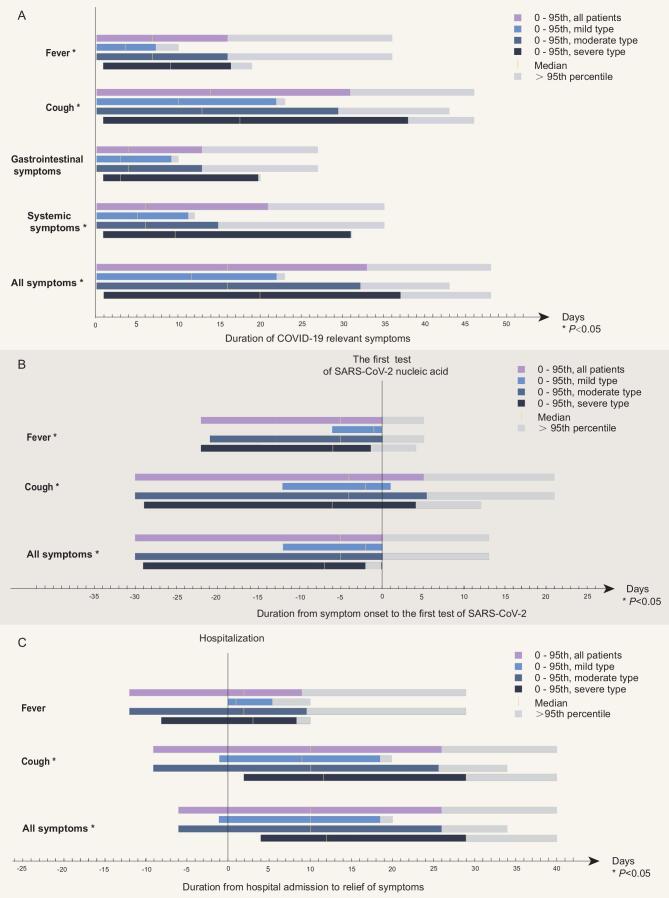
Dynamics of COVID-19 relevant symptoms in the study population. Figure 2 summarizes the dynamics of symptoms by the severity of COVID-19. (A) Overall, patients of more severe illness were prone to have longer duration of fever, coughing and systemic symptoms. For all patients, fever lasted for a median of 7.0 days (IQR 3.8 to 10.0 days), but coughing lasted significantly longer, for a median of 14.0 days (IQR 7.0 to 21.0 days). (B) The earliest onset of relevant symptoms presented a median of 5.0 days (IQR 2.8 to 8.0 days) before the screening test of SARS-CoV-2 RNA on detection, fever 5.0 days (IQR 2.0 to 8.0 days), and coughing 4.0 days (IQR 0 to 7.0 days). 3.26% (10/307) of the cases developed a fever after admission to the hospitals and 24.10% (74/307) developed coughing. (C) All COVID-19 symptoms lasted for a median of 10.0 days (IQR 6.0 to 15.0 days) after admission to the hospitals. But compared with cough (median 10.0 days, IQR 5.0 to 15.0 days) and other symptoms, fever lasted for a significantly shorter duration (median 2.0 days, IQR 0 to 4.0 days).

The median of the incubation period of all the investigated patients was 6.0 days (interquartile range [IQR] 3.0 to 10.0 days), and secondary cases had a slightly longer incubation of 7.0 days (4.0 to 12.5 days, *P* = 0.047, Table S1). The dynamics of symptoms by the severity of the disease were summarized in Fig. 2. The most common symptoms at initial assessment were fever (80.64%, 247/307) and coughing (62.87%, 193/307). As the main target of symptom-based surveillance, fever lasted for a median of 7.0 days (IQR 3.8 to 10.0 days), but coughing lasted significantly longer, for a median of 14.0 days (IQR 7.0 to 21.0 days) (Fig. 2A). Figure 2B showed that the earliest onset of relevant symptoms presented a median of 5.0 days (IQR 2.8 to 8.0 days) before the screening test of SARS-CoV-2 RNA on detection, fever 5.0 days (IQR 2.0 to 8.0 days), and coughing 4.0 days (IQR 0 to 7.0 days). 3.26% (10/307) of the cases developed a fever after admission to the designated hospitals, and 24.10% (74/307) developed coughing. Overall, in >90% of all the patients at least one relevant symptom could be detected before they received the screening viral RNA test.

All the patients were treated according to the recommendations in the *Diagnosis and Treatment Protocol for Novel Coronavirus Pneumonia* released by the National Health Commission of the People's Republic of China [15] (the National Protocol, see Methods). Details of treatments are available in the Supplementary Materials (Table S2). One patient died from COVID-19 in our study population. All other patients recovered from COVID-19 feeling approximately the same as before the disease. They were discharged based on the criteria noted in the National Protocol. At the end of the study period, 20 (6.51%) of them were finally classified as mild type COVID-19, 249 (81.11%) as moderate type and 38 (12.38%) as severe type (see Methods for criteria of classification).

### Clinical characteristics of asymptomatic patients on detection

Among all 307 cases, we identified 17 asymptomatic cases on detection, who presented with no relevant symptoms at the time of the first test of SARS-CoV-2 RNA before isolation. With regard to their exposure history, four of them had returned from Wuhan during the 14 days before hospitalization and 13 had close contact with confirmed COVID-19 cases (Fig. 1). Based on these data, the proportion of asymptomatic patients on detection was 5.54% (17/307), and the detection rate was 0.35% (13/3710).

As Table S3 shows, the 17 asymptomatic patients on detection were aged 41.46±17.12 years. Nine (52.94%) were in their middle ages (35 to 60 years). Ten (58.52%) were female. One (patient 6) had gout, and one (patient 14) had hypertension, others reported no previous comorbidities. Two of them were finally categorized as mild type, and 15 as moderate type, but none as severe type. No deaths were observed among these 17 patients.

To analyze the profile of these asymptomatic patients, we grouped the study population into four groups according to the presence of symptoms on detection: asymptomatic, afebrile but symptomatic, mild fever and moderate/high fever (Table S3). Compared with those afebrile but symptomatic, and those with mild or moderate/high fever on detection, asymptomatic patients on detection were more likely secondary cases (asymptomatic vs. afebrile vs. slight fever vs. moderate/high fever 76.47% vs. 51.16% vs. 26.92% vs. 22.38%, *P* = 0.001), less likely to progress into the severe type of COVID-19 (0% vs. 13.95% vs. 5.77% vs. 17.48%, *P* = 0.013), and apparently less likely to be admitted to the intensive care unit (0% vs. 4.65% vs. 4.81% vs. 13.29%, *P* = 0.057). A tendency of higher proportion of women (female%, asymptomatic vs. afebrile vs. slight fever vs. moderate/high fever, 58.52% vs. 60.47% vs. 37.50% vs. 36.36%, *P* = 0.013) and less coexisting disorders (11.76% vs. 11.63% vs. 20.19% vs. 23.08%, *P* = 0.366) were noted in asymptomatic and afebrile cases on detection.

During hospitalization, all these asymptomatic patients received antiviral treatments, largely similar to the other three groups (Table S2 and Table S4). But they were less likely to be in need of antibiotics and glucocorticoids. Laboratory test results were largely similar among the four groups (Table S3).

**Figure 3.**
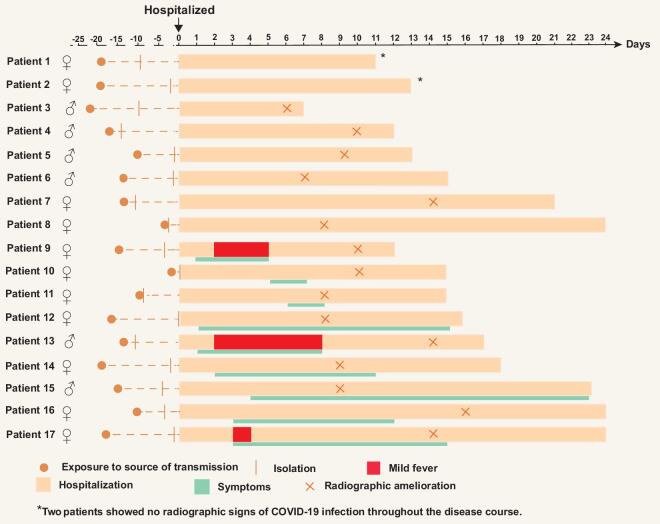
Dynamics of symptoms of patients of COVID-19 asymptomatic on detection. Figure 3 summarizes the dynamics of symptoms related to epidemiological history, hospitalization and change in their chest computed tomography imaging of the 17 patients asymptomatic when they first received the SARS-CoV-2 nucleic acid test. Eight of them presented with no symptoms throughout their disease course (Patients 1–8), and two of them showed no radiographic signs of COVID-19 infection at all (Patient 1 and 2). The rest of the nine patients (Patients 9–17) had a median incubation period of 17.0 days (IQR 14.0 to 19.0 days). All these 17 patients were hospitalized for a median of 15.0 days (IQR 13.0 to 21.0 days).

The median from admission to discharge of the asymptomatic patients on detection was 15.0 days (IQR 13.0 to 21.0 days) but could be as long as 23 days, which was similar to the other three groups. None of them had re-detectable viral nucleic acid during follow-up after discharge. Figure 3 demonstrates the dynamic of symptoms of 17 asymptomatic patients on detection. Eight patients were asymptomatic carriers who remained asymptomatic throughout their disease course, but only two of these carriers showed no signs of SARS-CoV-2 infection in their chest CT imaging throughout the disease course. Nine were presymptomatic on detection, and had symptoms developing later on despite receiving antiviral treatments after admission to the hospital, making their median incubation period 17.0 days (interquartile range, IQR 14.0 to 19.0 days) and their median duration of symptoms 9.0 days (IQR 4.0 to 12.0 days). The median duration of all 17 patients from the potential exposure to the source of transmission to admission was 15.0 days (IQR 14.0 to 18.0 days).

Interestingly, of the 176 patients (11 asymptomatic and 165 symptomatic on detection) tested for SARS-CoV-2 specific antibodies around 10 to 14 days of hospitalization, we found that the asymptomatic patients on detection had significantly lower levels of both IgG (cut-off index [COI], 2.60 [IQR 1.79 to 9.11] vs. 17.99 [IQR 5.23 to 40.72], *P* = 0.008) and IgM (COI, 0.78 [IQR 0.37 to 1.32] vs. 2.70 [IQR 0.99 to 6.80], *P* = 0.004) compared with those symptomatic on admission (COI above 1.2 defined as positive; Table 2).

**Table 2.** Levels of serum SARS-CoV-2 neutralizing antibodies in confirmed cases of COVID-19.

	All	Symptomatic patients	Asymptomatic patients	
	patients	on detection	on detection	*P* value

N	176	165	11	–
IgG, COI (IQR)	16.38(4.83,38.83)	17.99(5.23,40.72)	2.60(1.79,9.11)	0.008
IgG (positivity), n(%)	155(88.07)	145(87.88)	10(90.91)	1.000
IgM, COI (IQR)	2.43(0.97,6.51)	2.70(0.99,6.80)	0.78(0.37,1.32)	0.004
IgM (positivity), n(%)	114(64.77)	110(66.67)	4(36.36)	0.053
IgA COI (IQR)	4.11(1.41,10.07)	4.18(1.48,10.41)	2.93(0.98,5.46)	0.203
IgA (positivity), n(%)	138(78.41)	130(78.79)	8(72.72)	0.705

By analyzing the transmission and clustering data (Supplementary Materials, Fig. S1), We found that the asymptomatic patients on admission were in 11 cluster events involving 37 symptomatic patients and 17 asymptomatic patients on detection. Based on these cluster events, we estimated that one symptomatic patient would transmit the infection to a median of 1 person (IQR 0 to 2 persons; maximum eight persons), while the 17 asymptomatic patients transmitted the disease to no one, which is probably due to the swift and strict quarantine policy in these cities.

### Duration of the virus nucleic acid shedding asymptomatic phase during recovery

We found that in the convalescent patients of COVID-19, there was a phase in which their symptoms had been relieved, but the results for viral RNA tests in their sample were still positive. We investigated the duration of the virus RNA shedding asymptomatic phase, i.e. the interval of relief of all symptoms to the final negativity results of viral nucleic acid, in all 307 patients. The median duration of virus RNA shedding asymptomatic phase was 5.0 days (IQR 2.0 to 11.0 days) in all patients, 7.0 days (IQR 3.0 to 13.0 days) in mild type patients, 6.0 days (IQR 2.0 to 11.0 days) in moderate type patients and 4.0 days (IQR 1.0 to 9.0 days) in severe type patients (Table 3). Notably, 14 patients had re-detectable viral nucleic acid in the follow-up tests after discharge. For these 14 patients, the duration of virus RNA shedding asymptomatic phase could be up to 35.5 days (IQR 31.3 to 41.5 days).

## DISCUSSION

Asymptomatic infection of SARS-CoV-2 can be infectious and may be an important underlying cause of a pandemic [16]. There are three types of different asymptomatic status of COVID-19 infection: asymptomatic carriers, presymptomatic patients in their incubation period on detection and the asymptomatic phase in convalescent patients. In this population-based surveillance study, we found that asymptomatic carriers and presymptomatic patients on detection only accounted for a small proportion of the total SARS-CoV-2 infection in cities bordering Hubei during the epidemic between January and March. Under the stringent measures of social distancing, the transmission of the virus from asymptomatic patients was well controlled. Also, we found that for all the COVID-19 patients, after they turned asymptomatic, it took approximately one week for tests of viral nucleic acid to turn negative in samples from respiratory tracts.

### Characteristics and transmissibility of asymptomatic patients on detection

Overall, the proportion of asymptomatic patients on detection, including those asymptomatic carriers and presymptomatic patients, was 5.53% in our study, while 52.9% (9/17, overall 2.93%, 9/307) asymptomatic cases became symptomatic after their first viral RNA test. The proportion of asymptomatic infection was significantly higher in our study than that reported from China’s center for disease control (CDC) (5.53% vs. 1.2%) [17]. We also found that the proportion of presymptomatic patients on detection was higher than their counterparts among mild/moderate COVID-19 cases in Wuhan (2.93% vs. 1.63%) [18]. But a recent single center study outside Wuhan suggested the proportion of asymptomatic carriers was 4.4% [19], similar to our study. Of note, the detection rate of such asymptomatic infection among close contacts was similar to that in Iceland (0.35% vs. 0.57 to 0.8%), but the proportion of presymptomatic infections in our study was lower than Iceland (43%) [7] and a US nursing home (56%) [3]. This could be due to several reasons. The targeting testing strategy of the population with a high risk of SARS-CoV-2 infection in the Icelandic study and the relatively enclosed environment of the nursing home may overestimate the incidence of presymptomatic infections. Firstly, the data in our study were collected from a population-based surveillance system, which was under a more natural circumstance and minimized the number of missing cases. Moreover, in Anhui where our study was conducted, unprecedented strict measures were taken with regard to the public health response required since January 24, including massive surveillance, travel restriction, strict social distancing and universal facemask-wearing during necessary outings and cession of public events. These measures, which had been proved effective to contain the spread of the virus [20–22], contributed to reduce the transmission of the infection and reduce the number of asymptomatic infection cases.

All these asymptomatic patients received antiviral therapy after admission to the hospital. As shown in Table S3, such treatment did not significantly change their duration of hospitalization compared to those with symptoms on detection. Without a randomized trial, we could not determine whether the treatment had an impact on the development of symptoms.

Regarding transmissibility of the asymptomatic carriers and presymptomatic patients, some case reports or case series studies provided evidence that SARS-CoV-2 virus could spread from such patients [4,23,24]. A survey of 77 infector–infectee transmission pairs showed that 44% of secondary cases were infected during the index infection’s presymptomatic stage [25]. A study from Ningbo used a prospective design to follow up the viral load and clinical manifestations of 2147 close contacts of symptomatic and asymptomatic COVID-19 cases. They concluded that the virus infection rates of close contacts were 6.3% with symptomatic patients and 4.11% with asymptomatic patients, respectively [26]. A recent report analyzing the 455 contacts who were exposed to an asymptomatic virus carrier also suggested the infectivity of asymptomatic carriers might be low [27]. In fact, one patient among the 17 asymptomatic patients in our study (Patient 11) was breastfeeding her two-month old baby after she was infected with SARS-CoV-2, and she did not pass the infection to her baby. Due to the implementation of timely and effective public health interventions, we were not able to compare the transmission rates. However, based on the epidemiological data in our study, we can make a rough estimate that every asymptomatic patient on admission spread the disease to no one even under the same household, while a symptomatic onset patient could spread to a median of one secondary case and a maximum of eight. Collectively, this indicates that transmission from asymptomatic patients can be completely cut down with its lower infectivity and with proper disease control measures, such as the ones that were implemented in Anhui and all over China.

Besides, we also found a potential tendency for there to be a higher proportion of females among asymptomatic and afebrile cases on detection, which is consistent with the previous findings in Shanghai and Nanjing [4,24]. None of these asymptomatic patients on detection in our study progressed to the severe type or died, suggesting that they were of less risk of progressing into severe status. In contrast, symptomatic patients with higher fever on detection tend to have a shorter incubation period and increased risk of severe illness. One potential explanation would be patients asymptomatic on detection had a weaker immune response to the infection. Studies have shown that over-activated immune response could be the underlying causes of pulmonary inflammation and extensive pulmonary damage in severe cases, as well as progression and prognosis of the disease [28–30]. In our study we found that serum SARS-CoV-2 neutralizing antibodies and serum inflammatory cytokine interleukin-6 (Table 2 and Table S3) levels were significantly lower in the asymptomatic patients than those symptomatic on detection at the middle of their disease course, which indicated a lower level of immune activity and might explain the difference in disease prognosis. However, we did not have data of the dynamic change in these indices, and the results should be interpreted with caution.

**Table 3.** The duration of symptoms of the confirmed cases of COVID-19.

Symptoms	All patients	Mild type	Moderate type	Severe type	*P* values
Fever, days (IQR)	7.0(3.8,10.0)	3.5(2.0,5.0)	7.0(3.0,10.0)	9.0(7.3,11.8)	0.001
Cough, days (IQR)	14.0(7.0,21.0)	10.0(4.0,15.0)	13.0(6.0,20.0)	17.5(14.0,26.3)	0.001
Gastrointestinal symptoms, days (IQR)	4.0(2.0,7.0)	3.0(1.0,6.0)	4.0(2.0,6.5)	3.0(1.5,7.0)	0.782
Systemic symptoms, days (IQR)	6.0(2.0,10.0)	5.0(2.8,9.3)	6.0(2.0,10.0)	10.0(2.0,21.5)	0.021
All symptoms, days (IQR)	16.0(11.0,22.0)	11.5(7.0,14.0)	16.0(10.0,21.0)	20.0(16.0,29.0)	0.001

### Asymptomatic status during the convalescent stage

It is noteworthy that the asymptomatic status in the convalescent stage of a COVID-19 patient could last for seven days in mild type, and five days in moderate type, which accounted for over 80% of the total number of infections in our study. The guidelines among different countries and regions varied, but most of them suggested that patients could be discharged from the hospital when their symptoms were relieved, and they should continue home isolation for 14 days [31–34]. In China, COVID-19 patients were discharged only if their nucleic acid tests were negative for respiratory tract pathogen twice consecutively and were also asked to be isolated at home for 14 days after discharge [15]. But emerging evidence shows that patients with re-detectable viral nucleic acid after discharge is not uncommon [35,36]. Although re-detectable virus nucleic acid might be caused by the limitation of the RT-PCR method used for testing the viral RNA, and there is no evidence showing patients with re-detectable virus nucleic acid would spread the infection, it is necessary to reconsider the criteria for discharge and the duration of home isolation post-discharge. In our study, 14 of the patients (4.56%, 14/307) had re-detectable virus RNA during their follow-up. Most of such re-detectable positivity was within two weeks after discharge and turned negative within the next two weeks, and caused no new infection during home isolation. This proportion is similar to that reported by the Korean CDC (3.13%) [35]. These findings highlighted the importance of subsequent viral RNA tests during follow-up even if the patients are discharged with negative viral RNA results. But upon proper follow-up strategies and strict social distancing, these patients could be identified, and the risk of transmission could be minimized. Considering the time from symptom remission to viral nucleic acid was not short, and the issue of re-detectable positivity in our study, the current two-week and four-week follow-up schedule with virus tests after a viral negative discharge, is appropriate provided that testing capacity is sufficient.

### Fever was not enough as a solitary indicator for surveillance

Currently, temperature surveillance of fever at public places was the sole focus of infection-control strategies in some areas. In this study, we found that fever was neither the most frequent nor the symptom with the longest duration through our investigation of symptom dynamics: 83.71% of the cases had a fever at some point during their disease, but 86.97% of cases had coughing. Fever lasted for a significantly shorter time than coughing (7.0 days [IQR 4.0 to 10.0 days] vs. 14.0 days [IQR 7.0 to 19.5 days], Fig. 2A and C). Also, Figure 2B demonstrates that coughing, perhaps with a combination of other symptoms, could develop earlier than fever. These data indicated that using fever as the only indicator to identify SARS-CoV-2 potential infection is far from sufficient. But continuous massive surveillance or lockdown would be disastrous to the social-economic status and in turn harm the disease control implementation. Together with the two asymptomatic phases discussed above, close tracing of contacts of confirmed cases, strict social distancing, and universal facemask wearing would be a more feasible choice before an effective vaccine emerges.

### Strengths and limitations of this study

This is the first population-based surveillance study. Not only do we provide estimates for the proportion of COVID-19 patients in different asymptomatic phases, but also the dynamics of symptoms in different disease severity categories and their clinical outcomes. All these results provided evidence for developing strategies for disease control and treatment.

Our study has some limitations. Due to the limited sample size and the strict measures taken to contain the spread of the disease, we could not quantify the relative contributions of asymptomatic or presymptomatic patients to SARS-CoV-2 transmission. Secondly, the proportion of asymptomatic infection (including latent infection) was determined by population-based monitoring. The population-wide seroepidemiological survey has not yet been conducted. Therefore, further study is warranted to gain a better understanding.

## CONCLUSION

Asymptomatic carriers and presymptomatic patients only accounted for a small proportion of COVID-19. Massive surveillance and close contact tracing could help to detect these carriers and presymptomatic patients. Asymptomatic status in the convalescent stage of COVID-19 could last up to a week, indicating negativity in viral RNA is necessary as a de-isolation criterion, and that follow-up is recommended.

## METHODS

### Study oversight

This is an observational cohort study. This study is part of the project of ‘Construction of a bio-information platform for novel coronavirus pneumonia (COVID-19) patients follow-up in Anhui’ (ChiCTR2000030331). This study was approved by the institutional board of the First Affiliated Hospital of University of Science and Technology of China (2020-XG(H)-009).

### Population-based surveillance system

Since the domestic spread of SARS-CoV-2 in January 2020, strict precautionary measures have been implemented in Anhui province by the joint effort of the local governments, the CDCs, the health commissions and the communities. These measures included setting up ‘fever clinics’ dedicated to treating patients who presented with fever or any COVID-19 like symptoms [37]. In the fever clinics, all patients received tests for SARS-CoV-2 viral nucleic acid and chest CT scan. Any case with positivity in viral nucleic or chest CT abnormality would be admitted to designated hospitals for COVID-19 treatment and isolated in a single ward. Also, massive surveillance was implemented. The individuals fulfilling one of the following criteria were defined as suspected cases and were traced: (1) travel history to Wuhan or Hubei Province during the past 14 days; (2) wild animal exposure during the past 14 days; (3) presentation of COVID-19 like symptoms such as fever, dry coughing, dyspnea and diarrhea; (4) close contact with confirmed or suspected COVID-19 patients within two weeks of their disease onset; (5) other potentially suspected cases. All these individuals were identified by community or CDC staff in person or via telephone, subject to epidemiological investigation. They were home isolated for medical observation and had SARS-CoV-2 viral nucleic acid tests every three days. Similarly, any case with positivity in viral nucleic acid was admitted to the designated hospitals. Others continued to be observed until the 14th day. Such measures ensured that we were able to trace all the potential cases of SARS-CoV-2 infection. The procedures are depicted in the Supplementary Materials, Fig. S2.

### Study population

Based on the population-based surveillance system mentioned above, we acquired the information on the number of all cases that had been screened and confirmed in the cities of Anqing, Lu’an and Fuyang of Anhui Province, China, between 22 January and 16 April 2020. We chose these cities because they were Anhui cities most adjacent to the Hubei Province. In these cities, all the confirmed SARS-CoV-2 infected cases were admitted to the following three designated hospitals: Fuyang No. 2 People's Hospital, Anqing Hospital Affiliated to Anhui Medical University (Anqing Municipal Hospital), and Lu’an People's Hospital. We collected data and samples of these confirmed cases of SARS-CoV-2 infection that were hospitalized in these hospitals during the study period. The investigated individuals all agreed to participate in the study and provided written informed consent.

### Confirmation of SARS-CoV-2 infection

According to the National Protocol [15], SARS-CoV-2 infection was confirmed by positive results in throat swabs or respiratory specimens of real-time reverse transcription-polymerase chain reaction (RT-PCR) assay repeated twice using SARS-CoV-2 nucleic acid detection kits.

In Anhui province, to improve the quality of the detection, a two-step confirmation strategy was adopted. Samples of an individual were first tested in the laboratory of municipal CDC with two different detection kits. The municipal CDC laboratory crosschecked the results from the two kits to report a positive case. Then these positive samples were sent to the laboratory of Anhui Provincial CDC using the same procedure to test for the viral nucleic acid. If the positivity could be repeated in the provincial CDC laboratory, this case was finally confirmed as positive. All suspected and confirmed cases in Anhui Province were required to go through such a two-step confirmation.

### Data and sample collection

We collected epidemiological and clinical data of all confirmed cases in the participating hospitals onto case report forms adapted from International Severe Acute Respiratory and Emerging Infection Consortium (ISARIC)/World Health Organization (WHO) Clinical Characterization Protocol for severe emerging infections. Briefly, information on symptoms and disease onset, potential exposure to the pathogen, visits to healthcare facilities, hospitalization, treatment, pathogen and laboratory tests, and clinical outcomes, and follow-up visits were collected. Notably, for all the confirmed cases, we comprised a daily log at approximately the same time every morning to document the severity of their COVID-19 related symptoms based on the data extracted from the medical record.

Trained investigators collected information from the medical record system and uploaded it to the REDCap electronic data capture tools securely hosted at the Division of Life Science and Medicine, University of Science and Technology of China. A second investigator verified these records. Then a third investigator validated the data by crosschecking with the record in the medical record system and by communication with the physicians attending the individuals or telephone interviews with the individuals when necessary. Two independent licensed radiologists reviewed the original images of the chest CT scans. We relied on those reports which had consistent interpretation.

We collected serum samples from confirmed cases in the participating hospitals during their hospitalization.

### Classification and treatment

All the confirmed COVID-19 patients admitted to the participating hospital were attended according to the National Protocol [15]. They were classified into mild, moderate and severe type COVID-19 based on severity. Mild cases were defined as ‘the clinical symptoms were mild, and there was no sign of pneumonia on imaging’ in align with the National Protocol. Moderate cases were those ‘showing fever and respiratory symptoms with radiological findings of pneumonia’. Severe cases in our study were a combination of severe cases and critical cases defined by the National Protocol.

### Discharge and follow-up

A patient should meet all the following criteria according to the National Protocol to be discharged from the designated hospital. (1) body temperature returned to normal for more than three days; (2) significant improvement of respiratory symptoms; (3) significant improvement in pulmonary imaging; (4) samples from the respiratory tract were negative twice for SARS-CoV-2 nucleic acid (sampling interval being at least 24 hours).

After discharge, all patients were subjected to home isolation for another 14 days. We followed up these patients two weeks and four weeks after discharge. Of these patients, 14 had re-detectable viral nucleic acid in their nasopharyngeal swab samples during home isolation, and all turned negative at the end of the fourth week after discharge.

### Definitions

We defined the incubation period as the period between the earliest exposure to the potential transmission source of SARS-CoV-2 to the onset of illness (the presence of the earliest symptom).

Suspected cases were defined in alignment with the criteria used in the massive surveillance. Confirmed cases were patients who were confirmed as having the SARS-CoV-2 infection through the two-step protocol described above. Asymptomatic cases on detection were confirmed cases without presence of any relevant symptoms at the first test (i.e. screening test) of SARS-CoV-2 RNA, including two types of patients: asymptomatic carriers and presymptomatic patients on detection. Asymptomatic carriers, i.e. patients with asymptomatic infection, were confirmed cases without any relevant symptoms, and with/without a change in chest CT throughout their disease course (infection) until their SARS-CoV-2 RNA turned negative. Presymptomatic patients on detection were patients who were asymptomatic at their screening test of SARS-CoV-2 but later developed relevant symptoms during hospitalization. We defined the asymptomatic phase as the period of time when a confirmed case presented with no relevant symptoms, which could refer to the time between exposure to transmission source and the onset of symptom(s), or the time between overall symptom relief to the final conversion to negativity in viral RNA tests during recovery, or the duration of SARS-CoV-2 nucleic acid positivity in an asymptomatic carrier.

Based on the presence of fever on detection, we categorized the patients with symptomatic onset into three groups: afebrile, mild fever and moderate/high fever. Patients were considered afebrile on detection if their body temperature stayed under 37.3°C before the first test of SARS-CoV-2. Mild fever was defined as patients who had any record of body temperature above 37.3°C, but never higher than 38.0°C before detection. Moderate/high fever was defined as patients who had any body temperature recorded above 38.0°C before detection.

### SARS-CoV-2 specific antibody detection

Serum SARS-CoV-2 specific antibody levels were measured with chemiluminescent kits (Kangrun Biotech) for IgA (Doc no. KR/CE-01-B10, Revision A/0), IgG (Doc no. KR/CE-02-B10, Revision A/0) and IgM (Doc no. KR/CE-03-B10, Revision A/0). Briefly, the N-protein or receptor-binding domain (RBD) viral antigens were coated to magnetic particles to catch SARS-CoV-2 specific IgA, IgM and IgG in patient sera. Then a second antibody that recognized IgA, IgM or IgG was added for detection of IgA, IgM and IgG, respectively. The detected chemiluminescent signal over the background signal was calculated as relative light units (RLU). COI was the ratio of RLU to statistically determined cut-off (criterion). Then RLU was measured using a fully automatic chemical luminescent immunoanalyzer, Kaeser 1000 (Kangrun Biotech, Guangzhou, China).

### Statistical analysis

Continuous variables were presented as mean ± standard deviation or median (IQR). Comparisons between groups were performed with the Student's t-test, one-way ANOVA, or Mann-Whitney U test when appropriate. Categorical variables were presented as number (%) and compared using the χ^2^ test or Fisher's exact test when appropriate. A two-sided α of less than 0.05 was considered statistically significant. Data were analyzed using the R software, version 3.6.1 (R Foundation for Statistical Computing).

## Supplementary Material

nwaa141_Supplemental_File
