# Supplementary material for: Asymptomatic patients and asymptomatic phases of Coronavirus Disease 2019 (COVID-19): a population-based surveillance study
Source: Natl Sci Rev. 2020 Jun 23;7(10):1527–39. doi: 10.1093/nsr/nwaa141 (PMC7337770; doi:10.1093/nsr/nwaa141)
Supplement: nwaa141_Supplemental_File [file nwaa141_supplemental_file.docx]

**Supplemental Materials**

**Table of Contents**

[Table S1. Clinical and epidemiological characteristics of the confirmed cases of COVID-19: comparison between the cases detected in surveillance (primary cases) and the cases detected in close contact tracing (secondary cases). 1](#_Toc43554824)

[Table S2. Treatment of the confirmed cases of COVID-19 during hospitalization. 3](#_Toc43554825)

[Table S3. Clinical and epidemiological characteristics of the confirmed cases of COVID-19 by the presence of fever. 4](#_Toc43554826)

[Table S4. Treatment details of 17 asymptomatic patients on detection. 7](#_Toc43554827)

[Figure S1. Cluster events of 17 asymptomatic patients on detection. 8](#_Toc43554828)

[Figure S2. Massive surveillance system for COVID-19 in Anhui. 13](#_Toc43554829)

## Table S1. Clinical and epidemiological characteristics of the confirmed cases of COVID-19: comparison between the cases detected in surveillance (primary cases) and the cases detected in close contact tracing (secondary cases).

|  | **All patients** | **Primary cases** | **Secondary cases** | **P value** |
| --- | --- | --- | --- | --- |
| N | 307 | 132 | 175 |  |
| **Clinical and epidemiological Characteristics** | | | |  |
| Age, years | 42.90±14.57 | 40.60±12.83 | 44.63±15.57 | 0.016 |
| 0-14, n(%) | 8(2.61) | 2(1.52) | 6(3.43) |  |
| 15-49, n(%) | 203(66.12) | 101(76.52) | 102(58.29) |  |
| 50-64, n(%) | 76(24.76) | 24(18.18) | 52(29.71) |  |
| ≥65, n(%) | 20(6.51) | 5(3.79） | 15(8.57) |  |
| Male sex, n(%) | 180(58.63) | 86(65.15） | 94(53.71) | 0.058 |
| Current smoking, n(%) | |  |  | 1.000 |
| Current smoker | 27(8.79) | 12(9.09) | 15(8.57) |  |
| Former smoker or never smoked | 280(91.21) | 120(90.91) | 160(91.43) |  |
| Coexisting disorder, n(%) | 61(19.87) | 28(21.21) | 33(18.86) | 0.665 |
| Diabetes | 11(3.58) | 4(3.03) | 7(4.00) | 0.609 |
| Hypertension | 36(11.73) | 15(11.36) | 21(12.00) | 1.000 |
| Cardiovascular disease | 6(1.95) | 2(1.52) | 4(2.29) | 0.703 |
| Chronic pulmonary disease | 6(1.95) | 2(1.52) | 4(2.29) | 0.703 |
| Chronic liver disease | 13(4.23) | 9(6.82) | 4(2.29) | 0.013 |
| Chronic renal disease | 2(0.65) | 0 | 2(1.14) | 0.508 |
| Rheumatic disease | 3(0.98) | 2(1.52) | 1(0.57) | 0.579 |
| Symptoms, n (%) |  |  |  |  |
| Coughing | 193(62.87) | 86(65.15） | 107(61.14) | 0.638 |
| Sputum production | 98(31.92) | 51(38.64) | 47(26.86) | 0.103 |
| Hemoptysis | 2(0.65) | 1(0.76) | 1(0.57) | 1.000 |
| Sore throat | 15(4.89) | 9(6.82) | 6(3.43) | 0.238 |
| Snivel | 10(3.26) | 5(3.79） | 5(2.86) | 0.863 |
| Gasp | 6(1.95) | 1(0.76) | 5(2.86) | 0.440 |
| Dyspnea | 4(1.30) | 1(0.76) | 3(1.71) | 0.591 |
| Headache | 15(4.89) | 9(6.82) | 6(3.43) | 0.238 |
| Myalgia | 20(6.51) | 13(9.85) | 7(4.00) | 0.121 |
| arthralgia | 1(0.33) | 1(0.76) | 0 | 0.696 |
| Fatigue | 47(15.31) | 29(21.97) | 18(10.29) | 0.017 |
| Gastrointestinal symptoms | 23(7.49) | 11(8.33) | 12(6.86) | 0.666 |
| Severity classifications, n (%) |  |  |  | 0.037 |
| Mild | 20(6.51) | 9(6.82) | 11(6.29) |  |
| Moderate | 249(81.11) | 114(86.36) | 135(77.14) |  |
| Severe | 38(12.38) | 9(6.82) | 29(16.57) |  |
| Admission to ICU, n (%) | 27(8.79) | 9(6.82) | 18(10.29) | 0.391 |
| Median (IQR) time from onset of symptom to admission, days | 4.5(2.0,7.0) | 4.0(2.0,7.0) | 5.0(3.0,8.0) | 0.049 |
| Median (IQR) time from onset of symptom to discharge, days | 22.0(18.0,27.0) | 22.0(18.0,27.0) | 21.0(18.0,26.0) | 0.302 |
| Median (IQR) time from admission to discharge, days | 16.0(13.0,20.0) | 16.0(14.0,21.0) | 16.0(13.0,20.0) | 0.056 |
| Median (IQR) incubation period, days | 6.0(3.0,10.0) | 6.0(2.3,9.8) | 7.0(4.0,12.5) | 0.047 |
| **Laboratory findings on admission** (mean±SD unless otherwise noted) | | | |  |
| SaO_2_, % | 97.66±1.83 | 97.83±1.21 | 97.54±2.19 | 0.935 |
| White blood cell count, × 10^9^/L | 5.28±2.17 | 4.98±1.76 | 5.50±2.43 | 0.177 |
| ＜4 (leucopenia) ), n (%) | 93(30.29) | 42(31.82) | 51(29.14) | 0.339 |
| Neutrophilpercentage % | 65.24±13.34 | 64.96±12.14 | 65.45±14.23 | 0.578 |
| Lymphocyte percentage, % | 24.43±10.65 | 24.93±10.08 | 24.05±11.07 | 0.363 |
| ＜20 (lymphopenia), n (%) | 120(39.09) | 50(37.88) | 70(40.00) | 0.356 |
| Hemoglobin, g/L | 135.84±15.88 | 138.83±16.08 | 133.57±15.38 | 0.008 |
| Platelet count, × 10^9^/L | 184.82±76.11 | 172.05±59.90 | 194.56±85.35 | 0.059 |
| PT, s | 12.19±2.44 | 12.21±1.39 | 12.18±3.14 | 0.053 |
| APTT, s | 33.95±8.66 | 34.75±8.12 | 33.28±9.06 | 0.054 |
| ALT, U/L, median (IQR) | 24.00(15.00,37.00) | 27.00(15.75,42.00) | 23.00(14.75,34.00) | 0.024 |
| AST, U/L, median (IQR) | 25.00(20.00,32.00) | 31.38±27.30 | 29.15±22.58 | 0.109 |
| Total bilirubin, mmol/L | 13.29±7.74 | 12.33±7.62 | 14.04±7.76 | 0.036 |
| Creatinine, umol/L | 64.42±16.52 | 66.47±15.60 | 62.86±17.07 | 0.019 |
| BUN, mmol/L | 4.23±1.82 | 4.15±1.18 | 4.30±2.19 | 0.539 |
| Blood glucose, mmol/L | 6.42±2.11 | 6.49±2.11 | 6.37±2.12 | 0.811 |
| Procalcitonin, ng/mL, median (IQR) | 0.04(0.02,0.07) | 0.05(0.02,0.08) | 0.04(0.02,0.06) | 0.095 |
| C reactive protein, mg/L | 0.94±0.23 | 0.93±0.25 | 0.95±0.21 | 0.414 |
| CK, U/L, median (IQR) | 60.00(42.00,86.00) | 61.00(44.00,85.00） | 59.00(40.00,87.50) | 0.829 |
| CK-MB, U/L, median (IQR) | 7.00(3.00,11.00) | 6.00(3.75,10.25） | 7.00(3.00,12.00) | 0.854 |
| Interleukin 6, pg/ml, median (IQR) | 15.50(5.10,31.50） | 17.30(5.60,34.43) | 13.60(5.00,28.10) | 0.325 |
| Urinary protein (+ or ++), n(%) | 37(12.05) | 20(15.15) | 17(9.71) | 0.108 |
| Abnormalities in chest CT on admission, n (%) | 287(93.49) | 123(93.18) | 164(93.71) | 1.000 |

**Abbreviations:** IQR interquartile range, SaO2 Saturation of oxygen, PT prothrombin time, APTT activated partial thromboplastin time, ALT alanine aminotransferase, AST aspartate aminotransferase, BUN blood urea nitrogen, CK creatinine kinase, CK-MB creatinine kinase-MB.

## Table S2. Treatment of the confirmed cases of COVID-19 during hospitalization.

|  | **All patients** | **Asymptomatic on detection** | **Afebrile but symptomatic** | **Mild fever** | **Moderate/higher fever** | **P value** |
| --- | --- | --- | --- | --- | --- | --- |
| N | 307 | 17 | 43 | 104 | 143 |  |
| Antiviral Treatment, N (%) | 307(100) | 17(100) | 43(100) | 104(100) | 143(100) | 1.000 |
| Lopinavir and Ritonavir | 299(97.39) | 16(94.12) | 43(100.00) | 104(100.00) | 136(95.10) | 0.036 |
| Ribavirin | 32(10.42) | 3(17.65) | 10(23.26) | 10(9.62) | 9(6.29) | 0.011 |
| IFNα-2b | 238(77.52) | 16(94.12) | 36(83.72) | 75(72.12) | 111(77.62) | 0.157 |
| Neuraminidase inhibitor | 54(17.59) | 4(23.53) | 6(60.00) | 12(11.54) | 32(22.38) | 0.116 |
| Abidor | 73(23.78) | 4(23.53) | 9(20.93) | 27(25.96) | 33(23.08) | 0.926 |
| Antibiotics | 169(55.05) | 6(35.29) | 17(39.53) | 53(50.96) | 93(65.03) | 0.004 |
| Human Immunoglobulin | 39(12.70) | 0 | 2(4.65) | 4(3.85) | 33(23.08) | 0.001 |
| Corticosteroid | 78(25.41) | 2(11.76) | 6(13.95) | 22(21.15) | 48(33.57) | 0.015 |
| Chinese medicine | 269(87.62) | 16(94.12) | 40(93.02) | 94(90.38) | 119(83.22) | 0.214 |

## Table S3. Clinical and epidemiological characteristics of the confirmed cases of COVID-19 by the presence of fever.

|  | **All patients** | **Asymptomatic on detection** | **Afebrile but symptomatic** | **Slight fever** | **Moderate or high fever** | **P value** |
| --- | --- | --- | --- | --- | --- | --- |
| N | 307 | 17 | 43 | 104 | 143 |  |
| **Clinical and epidemiological Characteristics** | | | |  |  |  |
| Age, years | 42.90±14.57 | 41.46±17.12 | 45.18±13.16 | 42.63±14.57 | 42.58±14.75 | 0.676 |
| 0-14, n(%) | 8(2.61) | 1(5.88) | 0 | 2(1.92) | 5(3.50) | 0.021 |
| 15-49, n(%) | 203(66.12) | 7(41.18) | 27(62.79) | 75(72.12) | 94(65.73) |  |
| 50-64, n(%) | 76(24.76) | 9(52.94) | 13(30.23) | 16(15.38) | 38(26.57) |  |
| ≥65, n(%) | 20(6.51) | 0 | 3(6.98) | 11(10.58) | 6(4.20) |  |
| Male sex, n(%) | 180(58.63) | 7(41.18) | 17(39.53) | 65(62.50) | 91(63.64) | 0.013 |
| Current smoking, n(%) |  |  |  |  |  |  |
| Current smoker | 27(8.79) | 3(17.65) | 2(4.65) | 8(7.69) | 14(9.79) | 0.375 |
| Former smoker or never smoked | 280(91.21) | 14(82.35) | 41(95.35) | 96(92.31) | 129(90.21) | 0.375 |
| Exposure to source of transmission within the past 14 days, n(%) | | | |  |  |  |
| Recently visited Wuhan | 132(43.00) | 4(23.53) | 12(27.91) | 54(51.92) | 62(43.36) |  |
| Had contacted with confirmed patients | 94(30.62) | 13(76.47) | 22(51.16) | 28(26.92) | 32(22.38) |  |
| Not clear | 81(26.38) | 0(0.00) | 9(20.93) | 22(21.15) | 49(34.27) |  |
| Coexisting disorder, n(%) | 61(19.87) | 2(11.76) | 5(11.63) | 21(20.19) | 33(23.08) | 0.366 |
| Diabetes | 11(3.58) | 0 | 0 | 4(3.85) | 7(4.90) | 0.640 |
| Hypertension | 36(11.73) | 1(5.88) | 3(6.98) | 15(14.42) | 17(11.89) | 0.610 |
| Cardiovascular disease | 6(1.95) | 0 | 1(2.33) | 2(1.92) | 3(2.10) | 1.000 |
| Chronic pulmonary disease | 6(1.95) | 0 | 1(2.33) | 3(2.88) | 2(1.40) | 0.741 |
| Chronic liver disease | 13(4.23) | 0 | 0 | 4(3.85) | 9(6.29) | 0.812 |
| Chronic renal disease | 2(0.65) | 0 | 0 | 1(0.96) | 1(0.70) | 1.000 |
| Rheumatic disease | 3(0.98) | 1(5.88) | 0 | 1(0.96) | 1(0.70) | 0.293 |
| Symptoms, n (%) |  |  |  |  |  |  |
| Coughing | 193(62.87) | 0 | 34(79.07) | 65(62.50) | 94(65.73) | 0.001 |
| Sputum production | 98(31.92) | 0 | 10(23.26) | 36(34.62) | 52(36.36) | 0.001 |
| Hemoptysis | 2(0.65) | 0 | 0 | 1(0.96) | 1(0.70) | 0.796 |
| Sore throat | 15 (4.89) | 0 | 2(4.65) | 3 (2.88) | 10 (5.99) | 0.320 |
| Snivel | 10(3.26) | 0 | 3(6.98) | 5(4.81) | 2(1.40) | 0.130 |
| Gasp | 6(1.95) | 0 | 0 | 4(3.85) | 2(1.40) | 0.308 |
| Dyspnea | 4(1.30) | 0 | 0 | 0 | 4(2.80) | 0.362 |
| Headache | 15(4.89) | 0 | 3(6.98) | 6(5.77) | 6(4.20) | 0.737 |
| Myalgia | 20(6.51) | 0 | 4(9.30) | 6(5.77) | 10(6.99) | 0.507 |
| arthralgia | 1(0.33) | 0 | 0 | 0 | 1(0.70) | 0.297 |
| Fatigue | 47(15.31) | 0 | 8(18.60) | 19(18.27) | 20(13.99) | 0.256 |
| Gastrointestinal symptoms | 23(7.49) | 0 | 3(6.98) | 10(9.62) | 10(6.99) | 0.659 |
| Severity classifications, n (%) |  |  |  |  |  | 0.053 |
| Mild | 20(6.51) | 2(11.76) | 4(9.30) | 8(7.69) | 6(4.20) |  |
| Moderate | 249(81.11) | 15(88.24) | 33(76.74) | 89(85.58) | 112(78.32) |  |
| Severe | 38(12.38) | 0 | 6(13.95) | 7(6.73) | 25(17.48) |  |
| Admission to ICU, n (%) | 27(8.79) | 0 | 2(4.65) | 6(5.77) | 19(13.29) | 0.092 |
| Median (IQR) time from onset of symptom to admission, days | 4.5(2.0,7.0) | / | 4.0(2.0,8.0) | 4.0(2.0,7.0) | 5.0(3.0,7.0) | 0.594 |
| Median (IQR) time from onset of symptom to discharge, days | 22.0(18.0,27.0) | 16.0(10.0,19.0) | 22.0(17.5,26.5) | 21.0(18.0,28.0) | 22.0(18.0,26.5) | 0.011 |
| Median (IQR) time from admission to discharge, days | 16.0(13.0,20.0) | 15.0(13.0,21.0) | 15.0(13.5,19.0) | 16.0(13.0,20.0) | 16.0(13.0,20.0) | 0.913 |
| Median (IQR) incubation period, days | 6.0(3.0,10.0) | 17.0(14.0,19.0) | 8.0(3.0,15.0) | 6.0(2.3,9.0) | 5.0(3.0,10.0) | 0.001 |
| **Laboratory findings on admission** (mean±SD unless otherwise noted) | | | | | |  |
| SaO_2_, % | 97.66±1.83 | 97.88±0.86 | 97.67±1.92 | 97.87±1.09 | 97.49±2.27 | 0.557 |
| White blood cell count, × 10^9^/L | 5.28±2.17 | 5.76±2.35 | 5.27±1.74 | 5.30±1.91 | 5.20±2.45 | 0.414 |
| ＜4 (leucopenia) ), n (%) | 93(30.29) | 4(23.53) | 11(25.58) | 28(26.92) | 50(34.97) | 0.504 |
| Neutrophil percentage, % | 65.24±13.34 | 66.94±11.33 | 62.42±12.89 | 65.96±10.64 | 65.34±15.35 | 0.398 |
| Lymphocyte percentage, % | 24.43±10.65 | 23.50±11.09 | 27.74±11.23 | 23.97±8.84 | 23.88±11.52 | 0.230 |
| ＜20 (lymphopenia), n (%) | 120(39.09) | 7(41.18) | 14(32.56) | 39(37.50) | 60(41.96) | 0.079 |
| Hemoglobin, g/L | 135.84±15.88 | 134.82±12.70 | 134.79±18.79 | 135.53±16.03 | 136.52±15.27 | 0.477 |
| Platelet count, × 10^9^/L | 184.82±76.11 | 182.71±46.41 | 203.21±75.98 | 192.75±87.93 | 173.61±68.08 | 0.098 |
| PT, s | 12.19±2.44 | 11.50±0.65 | 12.88±5.38 | 11.99±1.33 | 12.22±1.45 | 0.258 |
| APTT, s | 33.95±8.66 | 33.55±6.29 | 33.90±13.20 | 33.32±7.53 | 34.44±7.96 | 0.676 |
| ALT, U/L, median (IQR) | 24.00(15.00,37.00) | 17.50(13.50,23.00) | 19.00(12.50,36.00) | 24.50(15.00,36.00) | 27.00(16.00,39.00) | 0.034 |
| AST, U/L, median (IQR) | 25.00(20.00,32.00) | 22.50(19.75,27.50) | 21.00(17.00,24.00) | 24.50(18.00,30.25) | 28.00(23.00,37.00) | 0.001 |
| Total bilirubin, mmol/L | 13.29±7.74 | 13.44±7.23 | 15.47±6.72 | 12.72±7.50 | 13.05±8.20 | 0.037 |
| Creatinine, umol/L | 64.42±16.52 | 58.98±14.28 | 59.86±13.20 | 67.28±18.11 | 64.29±16.07 | 0.071 |
| BUN, mmol/L | 4.23±1.82 | 4.69±1.77 | 4.26±1.44 | 4.36±2.38 | 4.08±1.39 | 0.616 |
| Blood glucose, mmol/L | 6.42±2.11 | 5.83±1.45 | 6.15±2.15 | 6.30±1.65 | 6.64±2.43 | 0.212 |
| Procalcitonin, ng/mL, median (IQR) | 0.04(0.02,0.07) | 0.04(0.03,0.05) | 0.04(0.02,0.06) | 0.04(0.02,0.06) | 0.05(0.02,0.08) | 0.295 |
| C reactive protein, mg/L | 0.94±0.23 | 1.00±0.01 | 0.93±0.26 | 0.96±0.19 | 0.93±0.26 | 0.515 |
| CK, U/L, median (IQR) | 60.00(42.00,86.00) | 59.00(42.50,76.25) | 50.00(37.00,61.00) | 55.00(43.00,80.00) | 66.00(44.00,97.50) | 0.036 |
| CK-MB, U/L, median (IQR) | 7.00(3.00,11.00) | 7.00(4.00,13.00) | 6.00(3.25,8.00) | 6.00(3.00,10.00) | 8.00(4.00,13.00) | 0.180 |
| Interleukin 6, pg/ml, median (IQR) | 15.50(5.10,31.50） | 4.50(3.40,12.43) | 4.60(3.85,16.00) | 12.00(4.93,23.53) | 21.00(9.75,42.70) | 0.001 |
| Urinary protein (+ or ++), n(%) | 37(12.05) | 0 | 3(6.98) | 13(12.50) | 21(14.69) | 0.794 |
| Abnormalities in chest CT on admission, n (%) | 287(93.49) | 15(88.24) | 39(90.70) | 96(92.31) | 137(95.80) | 0.285 |

**Abbreviations:** IQR interquartile range, SaO_2_ Saturation of oxygen, PT prothrombin time, APTT activated partial thromboplastin time, ALT alanine aminotransferase, AST aspartate aminotransferase, BUN blood urea nitrogen, CK creatinine kinase, CK-MB creatinine kinase-MB.

## Table S4. Treatment details of 17 asymptomatic patients on detection.

|  | **Patient type** | **Initiation to symptom onset (days)** | **Antiviral treatment**  **(Time from admission to initial day [days], duration [days])** | | | | |
| --- | --- | --- | --- | --- | --- | --- | --- |
|  |  |  | **Ribavirin** | **Lopinavir and Ritonavir** | **Aerosolized interferon α2b** | **Oseltamivir** | **Arbidol** |
| Patient 1 | carrier | n/a | / | 200mg/50mg bid p.o. (0, 6) | 5,000,000 IU bid (0, 10) | / | / |
| Patient 2 | carrier | n/a | / | 200mg/50mg bid p.o. (0, 11) | 5,000,000 IU bid (0, 11) | / | / |
| Patient 3 | carrier | n/a | / | / | 5,000,000 IU bid  (0, 7) | 75mg bid p.o.  (2, 5) | 0.2 tid p.o.  (0, 7) |
| Patient 4 | carrier | n/a | / | 200mg/50mg bid p.o. (1, 11) | / | / | / |
| Patient 5 | carrier | n/a | / | 200mg/50mg bid p.o. (0, 1) | 5,000,000 IU bid (0, 14) | / | / |
| Patient 6 | carrier | n/a | / | 200mg/50mg bid p.o. (0, 3) | 5,000,000 IU bid (0, 3) | / | / |
| Patient 7 | carrier | n/a | 0.5g q12h p.o.  (13, 8) | 200mg/50mg bid p.o. (1, 9) | 5,000,000 IU bid  (0, 19) | / | / |
| Patient 8 | carrier | n/a | / | 100mg/25mg bid p.o. (0, 15) | 5,000,000 IU bid  (3, 21) | / | / |
| Patient 9 | presymptomatic | 1 | / | 200mg/50mg bid p.o. (0, 6) | 5,000,000 IU bid  (0, 12) | 75mg bid p.o.  (2, 5) | 0.2 tid p.o.  (0, 12) |
| Patient 10 | presymptomatic | 4 | / | 200mg/50mg bid p.o. (1, 14) | 5,000,000 IU bid  (1, 2) | 75mg bid p.o.  (7, 2) | 0.2 tid p.o.  (3, 2) |
| Patient 11 | presymptomatic | 6 | / | 200mg/50mg bid p.o. (0, 4) | 30 μg bid  (0, 15) | / | / |
| Patient 12 | presymptomatic | 1 | / | 200mg/50mg bid p.o. (0, 5) | 30 μg bid  (0, 16) | / | / |
| Patient 13 | presymptomatic | 0 | / | 200mg/50mg bid p.o. (0, 10) | 5,000,000 IU bid  (0, 7) | / | / |
| Patient 14 | presymptomatic | 2 | / | 200mg/50mg bid p.o. (0, 11) | 5,000,000 IU bid  (0, 11) | / | / |
| Patient 15 | presymptomatic | 4 | 0.5g bid iv.  (17, 6) | 200mg/50mg bid p.o. (0, 16) | 5,000,000 IU bid  (0, 23) | / | 0.2 tid p.o.  (11, 12) |
| Patient 16 | presymptomatic | 3 | / | 200mg/50mg bid p.o. (0, 15) | 5,000,000 IU bid  (3, 12) | / | / |
| Patient 17 | presymptomatic | 3 | 0.5g q12h iv.  (17, 4) | 200mg/50mg bid p.o. (0, 10) | 5,000,000 IU bid  (0, 21) | 75mg bid p.o.  (0, 5) | / |

## Figure S1. Cluster events of 17 asymptomatic patients on detection.

**Event. A**

**Event. B**

**Figure S1. (Continued)**

**Event. C**

**Event. D**

**Event. E**

**Figure S1. (Continued)**

**Event. F**

**Event. G**

**Figure S1. (Continued)**

**Event. H**

**Event. I**

**Figure S1. (Continued)**

**Event. J**

**Event. K**

## Figure S2. Massive surveillance system for COVID-19 in Anhui.
